# Caveat Emptor: Commercialized Manganese Oxide Nanoparticles Exhibit Unintended Properties

**DOI:** 10.1021/acsomega.3c00892

**Published:** 2023-05-18

**Authors:** Celia Martinez de la Torre, Kasey A. Freshwater, Mara A. Looney-Sanders, Qiang Wang, Margaret F. Bennewitz

**Affiliations:** † Department of Chemical and Biomedical Engineering, Benjamin M. Statler College of Engineering and Mineral Resources, West Virginia University, Morgantown, West Virginia 26506, United States; ‡ Shared Research Facilities, West Virginia University, Morgantown, West Virginia 26506, United States

## Abstract

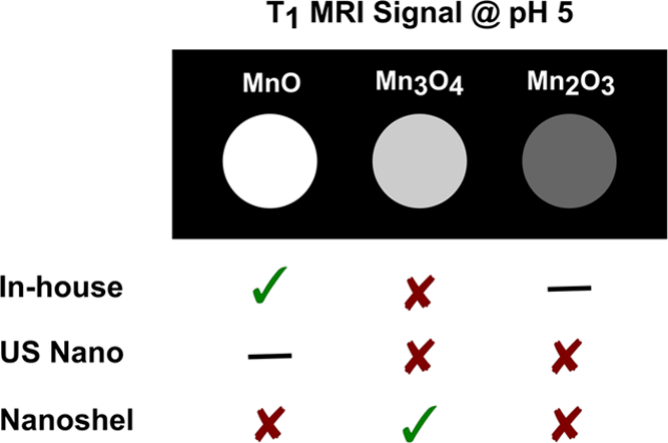

Nano-encapsulated manganese oxide (NEMO) particles are noteworthy contrast agents for magnetic resonance imaging (MRI) due to their bright, pH-switchable signal (“OFF” to “ON” at low pH), high metal loading, and targeting capability for increased specificity. For the first time, we performed a head-to-head comparison of NEMO particles from In-house and commercialized sources (US Nano vs Nanoshel) to assess their potential as bright T_1_ MRI contrast agents. Manganese oxide nanocrystals (MnO, Mn_2_O_3_, and Mn_3_O_4_) were systematically evaluated for size, chemistry, release of manganese ions, and MRI signal pre- and post-encapsulation within poly(lactic-*co*-glycolic acid) (PLGA). Suprisingly, a majority of the commercialized formulations were not as advertised by displaying unintended sizes, morphologies, chemistry, dissolution profiles, and/or MRI signal that precludes in vivo use. US Nano’s Mn_3_O_4_ and Mn_2_O_3_ nanocrystals contained impurities that impacted Mn ion release as well as micron-sized rodlike structures. Nanoshel’s MnO and Mn_2_O_3_ nanoparticles had very large hydrodynamic sizes (>600 nm). In-house MnO and Nanoshel’s Mn_3_O_4_ nanoparticles demonstrated the best characteristics with brighter T_1_ MRI signals, small hydrodynamic sizes, and high encapsulation efficiencies. Our findings highlight that researchers must confirm the properties of purchased nanomaterials before utilizing them in desired applications, as their experimental success may be impacted.

## Introduction

In approximately 40% of all magnetic resonance imaging (MRI) procedures, gadolinium-based contrast agents (GBCAs) serve as the gold standard by increasing both the signal and contrast.^1−3^ Unfortunately, due to their nonspecific accumulation in both benign and malignant tumors, GBCAs can lead to high false positive rates in certain cancers. Moreover, GBCAs can induce harmful side effects, such as nephrogenic systemic fibrosis in renally challenged patients and Gd-deposition in the brain, liver, skin, and bone.^4−8^ Thus, there has been a recent shift in research toward the use of metal oxide nanoparticles (NPs) because of their biocompatibility, biodegradability, and potential for surface functionalization.^9,10^

Metal oxides are multifaceted compounds utilized in numerous fields due to their advantageous electrical, photochemical, catalytic, and magnetic properties. In the case of manganese oxide, these nanocrystals (e.g., MnO, MnO_2_, Mn_2_O_3_, and Mn_3_O_4_) have been used for catalysis,^11−13^ energy storage,^14−16^ and water treatment.^17,18^ Recently, however, the uses of manganese oxide have extended to other applications within the biomedical field, such as biosensors^19,20^ (e.g., glucose and H_2_O_2_ detection) and MRI contrast agents.^21−23^ Compared to conventional MRI contrast agents, metal oxide NPs possess higher loading capacities and tunable surface properties that can lead to stronger MRI signals and longer blood circulation times.^24−26^

**Figure 1:**
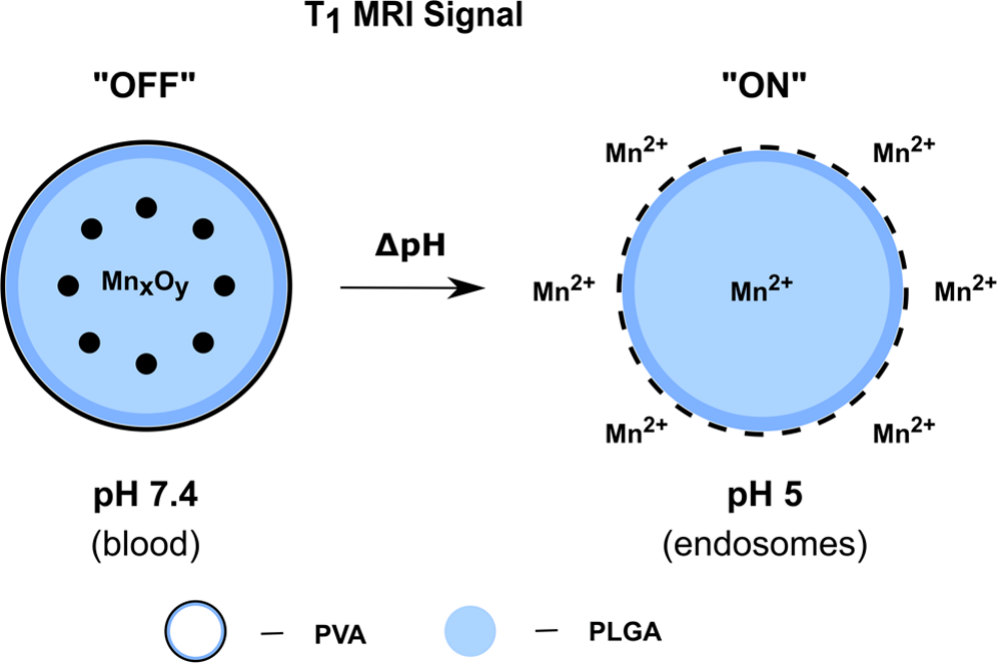
Schematic representation of the pH-switchability of NEMO particles. At low pH, NEMO particles dissolve to produce free manganese ions and turn “ON” the MRI signal.

To further study manganese’s efficacy as a potential replacement for GBCAs as a T_1_-based MRI contrast agent, we present poly(lactic-*co*-glycolic acid) (PLGA) nano-encapsulated manganese oxide (NEMO) particles, which possess several advantages.^10,27^ A significant limitation of GBCAs is their constantly active signal, which leads to contrast enhancement in normal tissues that can hide or mimic carcinomas depending on the intensity pattern.^28−31^ On the other hand, NEMO particles have a pH-switchable MRI signal that will convert from “OFF” to “ON” in low-pH environments (Figure 1), such as the endosomes (pH ∼5) in cancerous cells.^27,32^ This pH sensitivity in combination with their potential for functionalized NP targeting should increase the specificity of the signal in MRI. More specifically, targeting ligands on the NP surface will direct NEMO particles to malignancies reducing off-target effects.^33−35^ After entering the malignant tumor, NEMO particles will minimally dissolve in the extracellular space (pH 6.5). Once taken up by malignant cells due to the targeting ligand, NEMO particles enter acidic endosomes that will completely break down the polymer encapsulation, disassociate the manganese oxide complex, and release free manganese ions to produce a bright T_1_ MRI signal.^27,36,37^ The last significant advantage of our NEMO particles is their superior paramagnetism compared to GBCAs,^26,38^ which results in a brighter MRI signal.

As novel MRI contrast agents begin to approach clinical use, they must be synthesized in large quantities with reduced contamination and high purity. Lab-scale and commercialized NPs have their own advantages and disadvantages regarding cost effectiveness, scalability, contamination, shelf stability, and regulatory concerns.^39,40^ For example, with lab-scale operations, cross-contamination between other lab products is minimized compared to that of a large company charged with synthesizing numerous products using the same equipment. However, lab-scale synthesis is limited in batch size compared to industrial fabrication which produces much larger quantities.^41^ The bulk production, coupled with material changes such as oxidation, requires companies to set explicit shelf-life guidelines.^42^ Finally, the obtained purity of the desired phase of metal oxide can be greatly diminished based on starting materials and the synthesis technique alone, which are limiting factors for both operation types.^21^

For the first time, we present a systematic characterization of manganese oxide nanocrystals from In-house vs commercialized sources (US Research Nanomaterials (US Nano), and Nanoshel) pre- and post-encapsulation in PLGA to evaluate their use as T_1_ MRI contrast agents. Often, researchers will use these purchased nanomaterials in “as is” condition without verifying the products’ advertised properties,^43−45^ which if inaccurate, could unexpectedly affect experimental results. Thus, we sought to compare different manganese oxide NPs (MnO, Mn_2_O_3_, and Mn_3_O_4_) from In-house, US Nano, and Nanoshel for size, morphology, chemistry, loading, controlled release of manganese ions, and MRI signal.

Our results supported the Latin phrase “caveat emptor” or “let the buyer beware”, as a majority of the commercialized formulations were not as advertised, displaying unintended sizes, morphologies, chemistry, dissolution profiles, and/or MRI signal. In the case of US Nano, we discovered distinct impurities which impacted Mn ion release in addition to micron-sized and rodlike structures. Even though these encapsulated nanocrystals produced a bright T_1_ MRI signal at low pH for US Nano, their large, irregular size will likely prevent accumulation at the target site or even promote vessel occlusion. Although Nanoshel’s formulations had intended chemistries, the advertised sizes of some of their nanocrystals were not accurate and polymeric encapsulation of 2/4 nanocrystal types produced NPs too large for further in vivo applications. Thus, researchers are strongly encouraged to verify key properties of any purchased NPs prior to use; they may discover that In-house synthesis is the desirable method for their intended application. Overall, In-house MnO and Nanoshel’s Mn_3_O_4_ NPs presented with the best formulations based on their small hydrodynamic sizes, high encapsulation efficiencies, and brighter T_1_ MRI signals.

## Results and Discussion

In-house formulations of MnO and Mn_3_O_4_ nanocrystals were systematically compared to commercially available MnO, Mn_2_O_3_, and Mn_3_O_4_ nanocrystals purchased from two different companies (US Nano and Nanoshel) to evaluate their effectiveness as MRI contrast agents. As previously described, our In-house MnO and Mn_3_O_4_ formulations were synthesized via thermal decomposition of Mn(II)AcAc with oleylamine as the capping agent.^36,37,46^ Bare nanocrystals were characterized for crystal structure, chemical composition, impurities, size, morphology, and coating. Hydrophobic bare nanocrystals were encapsulated within PLGA and characterized for surface chemistry, size, morphology, encapsulation efficiency, pH-sensitive release of manganese ions, and resulting MRI signal. Throughout the manuscript, nanocrystals refer to bare manganese oxides, whereas NPs refer to PLGA-encapsulated manganese oxides.

### Chemical Composition of In-House vs Commercialized Manganese Oxide Nanocrystals

**Figure 2:**
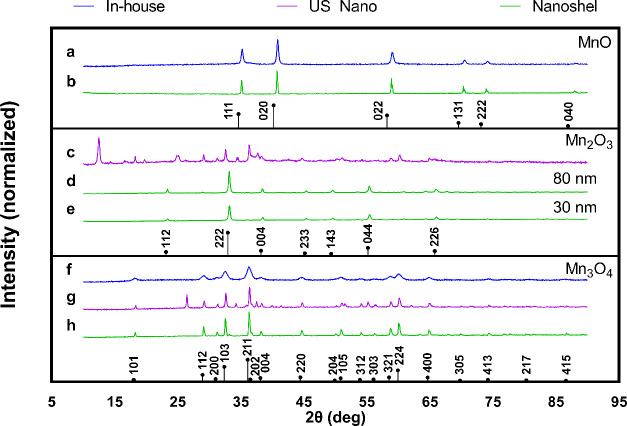
XRD spectra for (a) In-house MnO, (b) Nanoshel’s MnO, (c) US Nano’s Mn_2_O_3_, (d) Nanoshel’s Mn_2_O_3_-80 nm, (e) Nanoshel’s Mn_2_O_3_-30 nm, (f) In-house Mn_3_O_4_, (g) US Nano’s Mn_3_O_4_, and (h) Nanoshel’s Mn_3_O_4_ nanocrystals. Standard diffraction peaks (black) and corresponding samples are shown for MnO (top), Mn_2_O_3_ (middle), and Mn_3_O_4_ (bottom). Miller indices are indicated above the standard diffraction peaks. Note: A small shift can be observed between the standard MnO diffraction peaks compared to the In-house MnO and Nanoshel’s MnO nanocrystal peaks due to slight sample misalignment. US Nano’s Mn_2_O_3_ nanocrystals had calcium and sodium impurities, which were confirmed with additional XRD spectra (Supporting Figure S1) and SEM-EDS (Supporting Figures S2 and S3).

For bare In-house and commercially available manganese oxide nanocrystals, the crystal structure and chemical composition were verified using the gold-standard technique, X-ray diffraction (XRD). This technique confirmed the correct crystal structure of the In-house and Nanoshel’s manganese oxide nanocrystals, with 100% purity (Figure 2 and Table S1). Nanoshel’s other samples also exhibited high purity ∼98–99%. However, in the case of US Nano, additional characteristic peaks were found during analysis that could be attributed to other types of crystals. For US Nano’s Mn_2_O_3_ nanocrystals, peaks indicated a composition of 55.7% Mn_3_O_4_, 39.3% sodium birnessite, 3.6% calcium hexamanganese(III) manganese(IV) dodecaoxide, and 1.4% silicon dioxide; surprisingly, there were no peaks attributed to Mn_2_O_3_, the intended compound. US Nano’s Mn_3_O_4_ nanocrystals included 30.9% manganite (HMnO_2_) (Supporting Figure S1). These impurities could have been a result of cross-contamination from the synthesis equipment^40^ or the use of an incorrect starting material for the specific synthesis method.^21^ Although both companies marketed their nanocrystals with high purities >99%, only Nanoshel’s nanocrystals were close to the advertised specifications.

To further investigate the impurities found in US Nano’s materials, scanning electron microscopy–energy-dispersive X-ray spectroscopy (SEM-EDS) was performed on all of the nanocrystals (Supporting Figure S2). In the case of US Nano’s Mn_2_O_3_, additional elements including sodium and calcium were found to be present in the sample aside from manganese and oxygen. A mapping scan was conducted to assess if the impurities were associated with specific areas of the nanocrystals. As shown in Supporting Figure S3, the impurities, especially sodium, were spread homogeneously around the nanocrystals. US Nano’s Mn_3_O_4_ only showed the presence of manganese and oxygen in both the spectrum and mapping scans (Supporting Figures S2 and S3). Although the results indicate the presence of only manganese and oxygen, it is essential to highlight that one of the main limitations of SEM-EDS is the inability to detect hydrogen;^47,48^ therefore, HMnO_2_ could still be present, but could not be identified with spectroscopy.

### Size and Morphology of In-House vs Commercialized Nanocrystals

**Figure 3:**
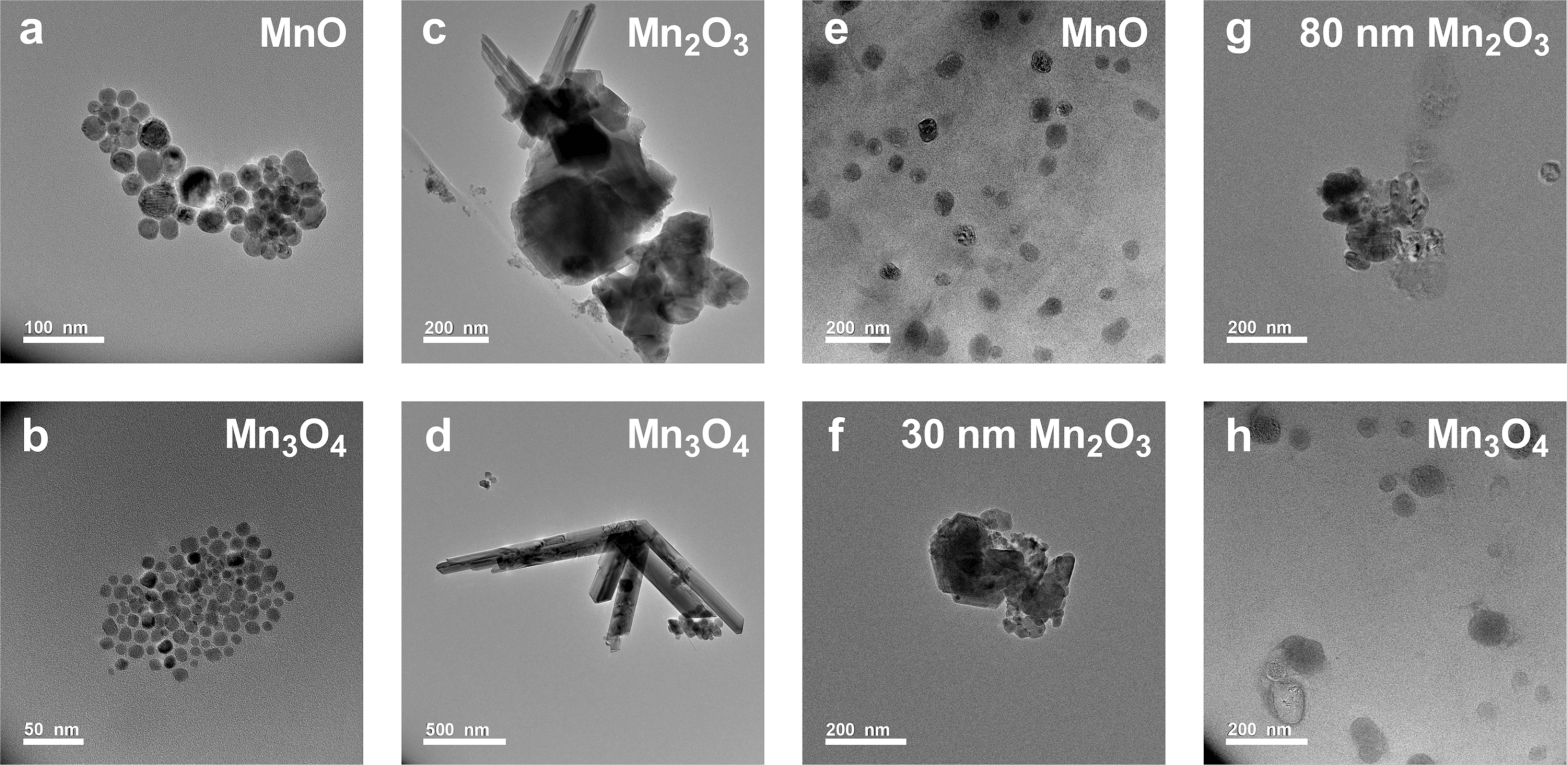
TEM images of In-house (a) MnO and (b) Mn_3_O_4_; US Nano’s (c) Mn_2_O_3_ and (d) Mn_3_O_4_; and Nanoshel’s (e) MnO, (f) Mn_2_O_3_-30 nm, (g) Mn_2_O_3_-80 nm, and (h) Mn_3_O_4_ nanocrystals. Elongated rodlike morphologies were found interspersed within US Nano’s nanocrystals, while all other nanocrystals displayed octagonal to round shapes.

**Figure 4:**
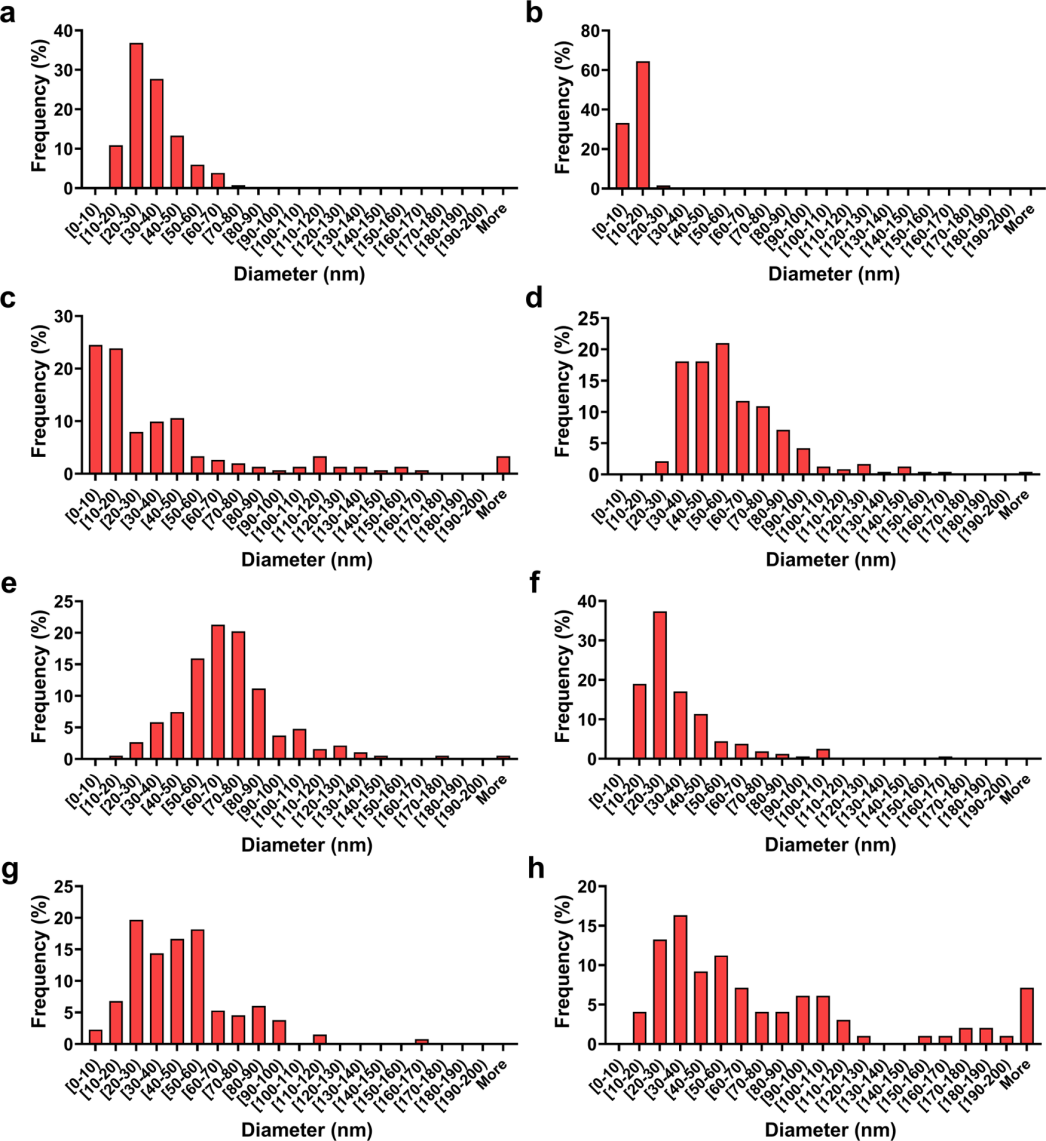
TEM size distribution of In-house (a) MnO and (b) Mn_3_O_4_; US Nano’s (c) Mn_2_O_3_ and (d) Mn_3_O_4_; and Nanoshel’s (e) MnO, (f) Mn_2_O_3_-30 nm, (g) Mn_2_O_3_-80 nm, and (h) Mn_3_O_4_ nanocrystals. Comparatively, In-house nanocrystals have a narrower size distribution compared to commercialized nanocrystals. Only round-shaped nanocrystals are considered in the above histograms of nanocrystal diameters.

NP size is another essential characteristic to control during synthesis of metal oxide NPs to maximize loading and controlled release of manganese ions for enhanced MRI signal. XRD spectra provided qualitative information on the difference in size based on the Scherrer equation, where broader peaks represent smaller nanocrystals.^11,49^ Based on the XRD spectra, Nanoshel’s nanocrystals were the largest, while In-house nanocrystals were the smallest. In addition, transmission electron microscopy (TEM) was used to quantify and compare the size of the manganese oxide nanocrystals in more detail, as shown in Figures 3 and 4. In the case of In-house nanocrystals, MnO and Mn_3_O_4_ showed an octahedral to round shape with an average size of 34 ± 13 and 11 ± 4 nm, respectively (Figure 3a,b).

Though the sizes advertised for both US Nano’s Mn_2_O_3_ and Mn_3_O_4_ were 30 nm (Table 1), both samples had two distinct populations formed: a rod-shaped group and a round-shaped group, which were measured separately. Due to the majority of nanocrystals being round-shaped (∼90% round vs ∼10% rod) for US Nano’s Mn_2_O_3_ and Mn_3_O_4_, only diameters for the round-shaped nanocrystals are shown in Table 1 and in Figure 4. In the case of the round-shaped nanocrystals, the diameters were 43 ± 53 and 61 ± 27 nm for Mn_2_O_3_ and Mn_3_O_4_, respectively (Figure 3c,d). US Nano’s nanocrystals had a broad distribution causing the high standard deviation of the size assessment (Figure 4). On the other hand, the rod-shaped nanocrystals ranged in length between 100 nm and 1.5 μm (median ∼436 nm) and 50 nm and 1.2 μm (median ∼169 nm) for Mn_2_O_3_ and Mn_3_O_4_, respectively. The presence of the rod and round structures can be attributed to variations in the synthesis technique, as both thermal decomposition and hydrothermal methods can create all three types of manganese oxides.^21^ For example, using a different starting material such as manganese oleate via thermal decomposition^50^ or manganese(II) nitrate via hydrothermal decomposition^51^ can result in either rod-shaped manganese oxide nanocrystals or a mixture of round- and rod-shaped nanocrystals, respectively.

**Table 1:** Marketed vs Actual Nanocrystal Size Determined by TEM, Shown as Average +/– St. Dev^a^

		nanocrystal size (nm)		
nanocrystal type		marketed	TEM	%error
In-house	MnO	n/a	34 ± 13	n/a
	Mn_3_O_4_	n/a	11 ± 4	n/a
US Nano	Mn_2_O_3_	30	43 ± 53	43
	Mn_3_O_4_	30	61 ± 27	103
Nanoshel	MnO	≤80	71 ± 29	0
	Mn_2_O_3_-30 nm	30	35 ± 22	17
	Mn_2_O_3_-80 nm	80	47 ± 25	41
	Mn_3_O_4_	10–20	77 ± 60	285

a %Error is experimental size compared to marketed size.

Moreover, Nanoshel’s marketed size was ≤80 nm for MnO, 30 nm for Mn_2_O_3_-30, 80 nm for Mn_2_O_3_-80, and 10–20 nm for Mn_3_O_4_ as shown in Table 1. TEM analysis determined that sizes for MnO (71 ± 29 nm) and Mn_2_O_3_-30 nm (35 ± 22 nm) were close to their intended target sizes. However, Mn_2_O_3_-80 nm (47 ± 25 nm) was 41% smaller than Nanoshel’s advertised size of 80 nm, whereas Mn_3_O_4_ (77 ± 60 nm) was 285% larger than the marketed size of 10–20 nm. As size variation can negatively impact subsequent experiments, the nanocrystal diameter should always be assessed prior to moving forward. It is essential to highlight that Nanoshel’s MnO and Mn_3_O_4_ had an unknown film that complicated our imaging efforts of the nanocrystals as observed in Figure 3e,h. It was unclear if the film was associated with the capping of the nanocrystals or residual reagents from the synthesis, meaning that further analysis was required.

### Surface Coating of In-House vs Commercialized Nanocrystals

First, Fourier transform infrared spectroscopy (FTIR) was used on all of the nanocrystals to assess the coating (Supporting Figure S4). As previously reported, an oleylamine coating was found on the In-house nanocrystals.^36,37,46^ For all of Nanoshel’s nanocrystals, the main highlight was the high presence of a broad peak in the 4000 to 1100 cm^–1^ frequency range, which could be indicative of a complex mixture of organic compounds or functional groups.^52^ Detection of these specific organic compounds is a goal of future work. Thermogravimetric analysis (TGA) was utilized to quantify the amount of coating found on the nanocrystals (Supporting Figure S5). TGA revealed that the In-house MnO and Mn_3_O_4_ had an oleylamine coating of approximately 10 and 17%, respectively. Both of Nanoshel’s Mn_2_O_3_ nanocrystals exhibited a small amount of coating (<3.5% weight) through sample burn-off above 700 °C. However, no change in sample weight was observed up to 800 °C for Nanoshel’s MnO and Mn_3_O_4_ nanocrystals. Thus, it is unclear what constituted the film observed on TEM which obscured imaging for Nanoshel’s MnO and Mn_3_O_4_ samples, as most functional groups decompose with heating prior to 800 °C. One of the main challenges in working with commercialized samples is the lack of knowledge of the synthesis process and starting materials, which can complicate analysis.

### Size and Morphology of In-House vs Commercialized Manganese Oxide PLGA NPs

**Figure 5:**
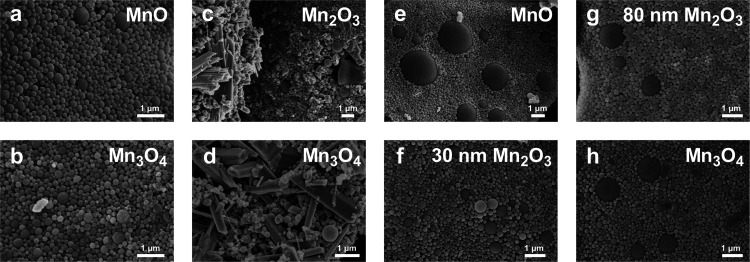
SEM images of PLGA-encapsulated In-house (a) MnO and (b) Mn_3_O_4_ NPs; US Nano’s (c) Mn_2_O_3_ and (d) Mn_3_O_4_ NPs; and Nanoshel’s (e) MnO, (f) Mn_2_O_3_-30 nm, (g) Mn_2_O_3_-80 nm, and (h) Mn_3_O_4_ NPs. Note the long rod-shaped structures present in US Nano’s samples along with round-shaped NPs; In-house and Nanoshel’s NPs only had spherical shapes.

For hydrophobic, inorganic manganese oxide nanocrystals to be used as contrast agents, they must be made hydrophilic. Herein, the nanocrystals were rendered hydrophilic through encapsulation within 7.5K-PLGA via a single emulsion technique. As shown by SEM (Figures 5 and S6) and FTIR spectroscopy (Supporting Figure S7), PLGA encapsulation was successful to generate spherical NPs with characteristic PLGA peaks for In-house and Nanoshel’s NPs. On the other hand, SEM images for US Nano (Figures 5c,d and S6) showed rod-shaped nanocrystals with the same morphology and size as presented by TEM (Figure 3c,d) in addition to large round-shaped NPs. The large size of these rod-shaped structures could promote higher amounts of proteins to bind upon injection into the bloodstream, causing faster elimination through the liver and spleen to reduce the circulation time and subsequent contrast agent accumulation in the desired area.^53^

**Figure 6:**
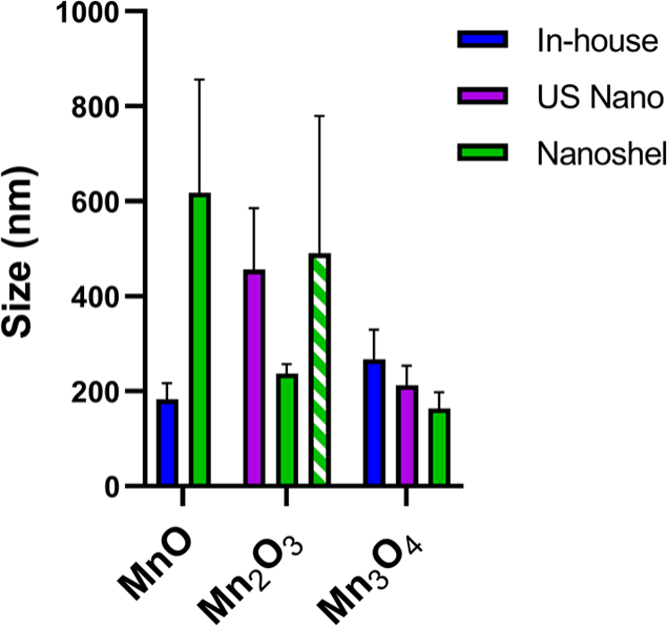
Average hydrodynamic size determined via DLS for PLGA-encapsulated In-house (blue), US Nano’s (purple), and Nanoshel’s (green) MnO, Mn_2_O_3_, and/or Mn_3_O_4_ NPs. Note that Nanoshel’s Mn_2_O_3_-30 nm is shown in solid green and Mn_2_O_3_-80 nm is shown in striped green. Average size is plotted with standard error of the mean; no significance was detected.

For Nanoshel’s PLGA MnO, Mn_2_O_3_-80 nm, and Mn_3_O_4_ NPs, two distinct size populations were present: a nanoscale one and a microscale one (Figures 5e,g,h and S6). A possible reason for the two populations is the broad size distribution of the bare nanocrystals as observed on TEM (Figure 4). Although SEM is an excellent technique to evaluate morphology, it cannot provide insight into how the NPs would interact in the body. Assessment of the hydrodynamic diameter using dynamic light scattering (DLS) is a more accurate analysis of size, as it can evaluate how an aqueous solution alters the NP size and if suspension promotes NP aggregation. Despite these advantages, DLS cannot measure the NP size larger than 10 μm, precluding measurements of any larger particles or aggregates.^54^Figure 6 and Supporting Table S2 and Figures S8–S10 show the results from DLS analysis of the PLGA-encapsulated manganese oxides. Most NPs had a hydrodynamic size of approximately 200–250 nm, except for US Nano’s Mn_2_O_3_, Nanoshel’s MnO, and Nanoshel’s Mn_2_O_3_-80 nm, which were larger. DLS has some additional limitations that could impact the diameter measurements including (1) sedimentation by dense NPs, which could be prevented by using stabilizers such as sucrose, (2) sample concentration where higher amounts of NP samples can lead to multiple light scattering interactions between closely spaced NPs, and (3) high scattering intensity from large NP aggregates, which will dominate measurements even if present in small quantities.^55,56^

Size is a critical factor in contrast agent design, as it determines where NPs travel, accumulate, and are eliminated in the body. For example, NPs with a hydrodynamic size below 5 nm prefer accumulation in the kidney and are excreted in the urine, while larger NPs favor the liver and spleen and will be eliminated in the feces.^57^ Since the goal of a contrast agent would be to accumulate within the tumor, it is necessary to take into consideration the leakiness of the tumor vasculature and the poor lymphatic drainage, also known as the enhanced permeability and retention (EPR) effect.^58−60^ Based on the literature, an ideal size would be between 50 and 200 nm to ensure adequate tumor penetration and retention,^57,61,62^ which is similar to a majority of the NEMO particles synthesized herein.

### pH-Dependent Mn^2+^ Release from In-House vs Commercialized Manganese Oxide PLGA NPs

**Figure 7:**
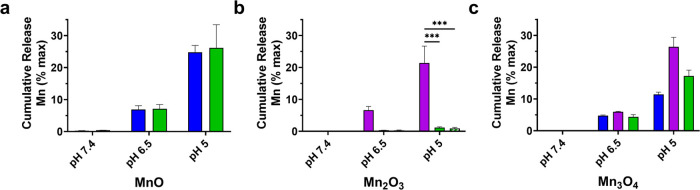
Average cumulative release of Mn^2+^ from PLGA-encapsulated (a) MnO, (b) Mn_2_O_3_, and (c) Mn_3_O_4_ NPs after 1 h of incubation at pH 7.4, pH 6.5, and pH 5. In-house nanocrystals are shown in blue, US Nano’s nanocrystals are displayed in purple, and Nanoshel’s nanocrystals are shown in green (striped bar represents Mn_2_O_3_-80 nm). Note the maximal Mn^2+^ release at pH 5 mimicking cell endosomes/lysosomes. Average release is plotted with standard error of the mean; statistical comparison was performed using two-way ANOVA with Holm–Šídák correction. *P* values are reported as * ≤0.05, *** ≤0.005.

Following encapsulation, testing the release of free manganese ions at different pH levels was necessary to evaluate their properties as future MRI contrast agents. As previously mentioned, manganese oxide nanocrystals are pH-sensitive. From our previous work, we anticipated minimal release of Mn^2+^ at neutral pH 7.4 mimicking blood, low release of Mn^2+^ at pH 6.5 mimicking the tumor extracellular space, and maximal release at pH 5 mimicking cellular endosomes/lysosomes for all formulations.^27,36^ As shown in Figures 7 and S11, all of the NPs had a negligible release at pH 7.4 after 1 h; however, as the pH became increasingly acidic, some NPs started to show the release of free manganese ions.

Both encapsulated In-house and Nanoshel’s MnO represented the highest manganese release after 1 h of ∼25% at pH 5, although there was not a statistical difference between them. For Mn_2_O_3_, US Nano had a statistically higher release compared to Nanoshel’s PLGA Mn_2_O_3_ NPs (21% vs <2% at pH 5, respectively). As shown in Supporting Table S1, US Nano’s Mn_2_O_3_ was comprised of a majority of Mn_3_O_4_ (55.7%), which dissolves at a faster rate than Mn_2_O_3_ and will produce more Mn^2+^ ions,^63^ as explained in more detail below. When analyzing the PLGA Mn_3_O_4_ NP cumulative release, once again, US Nano had a significantly higher release (26% at pH 5). When comparing Nanoshel’s and In-house PLGA Mn_3_O_4_ NPs (17% vs 11% at pH 5, respectively), no statistical difference was observed, but Nanoshel had a slightly higher release after 1 h. Moreover, Nanoshel had a smaller hydrodynamic diameter (164 vs 267 nm, Supporting Table S2), increasing the surface area-to-volume ratio and subsequently increasing the release rate of manganese at 1 h at pH 5. Regarding encapsulation efficiency shown in Supporting Table S2, there was no statistical difference discerned between any experimental group. However, when considering significance in Mn cumulative release, encapsulation efficiencies greater than 70% displayed a higher release of Mn compared to those that did not. The obtained NP yield across all formulations ranged from 43 to 59% (Supporting Table S2).

To compare manganese oxide varying crystalline structures between distinct chemistries, it is necessary to consider how each chemical composition dissociates differently. For MnO, in acidic environments, it directly dissociates into Mn^2+^ as shown by eq 1. Meanwhile, Mn_2_O_3_ and Mn_3_O_4_ dissociate into MnO_2_ and Mn^2+^ as per eqs 2 and 3, resulting in incomplete dissolution of the metal oxides; MnO degrades at a faster rate in acidic solutions compared to Mn_3_O_4_, which degrades more quickly than Mn_2_O_3_.^63^ Thus, to complete dissolution, Mn_2_O_3_ and Mn_3_O_4_ follow eqs 4 and 5 after primary dissolution. As a result, the manganese ion release follows the trend MnO > Mn_3_O_4_ > Mn_2_O_3_.
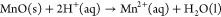
1
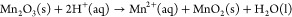
2
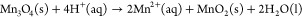
3
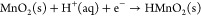
4
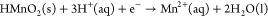
5The described trend was observed in all NPs except for US Nano’s Mn_2_O_3_ and Mn_3_O_4_ NPs, which displayed ≥95% release at pH 5 after 24 h. The impurities that US Nano has shown were the main contributors to this discrepancy. For Mn_2_O_3_, the presence of Mn_3_O_4_ would cause an immediate boost to Mn^2+^ release, as Mn_3_O_4_ dissociates faster than Mn_2_O_3_; the impact of the other impurities on release rate is unclear. For Mn_3_O_4_, the main impurity found was HMnO_2_, which, as shown by eq 5, would also promote faster Mn^2+^ release.^64^ Due to their slow release, Mn_2_O_3_-based contrast agents would require a longer waiting time between the administration and MRI scan, as the signal originates from free Mn^2+^. Even after 24 h (Supporting Figure S11), barely any manganese has been released (<13% at pH 5) from PLGA-encapsulated Mn_2_O_3_ NPs from Nanoshel. The explained timeline is not clinically relevant and would make the Mn_2_O_3_-based contrast agents not suitable.

When considering NEMO particles as alternative contrast agents to GBCAs, Mn toxicity will need to be assessed, as free Mn^2+^ ions will be released in low-pH intracellular endosomes. Even though Mn^2+^ is less toxic than Gd^3+^, free Mn^2+^ ions mimic Ca^2+^ and can enter neurons and muscles. In fact, free Mn^2+^ has been used for manganese-enhanced MRI (MEMRI) to visualize neuronal activity safely in rats up to a cumulative dose of 180 mg/kg Mn over 12 days.^65^ Recent studies have shown that MnO NPs themselves are well tolerated in vivo with no negative effects in mice^26,66^ at doses up to 20 mg/kg or in rats^67^ at 35 mg/kg Mn over acute time frames; Mn cleared from the vital organs in 24 h, with Mn levels in the brain matching saline controls.^67^ Future studies should evaluate chronic toxicity of NEMO particles in vivo for any hepatic, cardiac, and sensorimotor effects in healthy and tumor-bearing animals to ensure long-term biocompatibility.

### pH-Dependent T_1_ MRI Signal of In-House vs Commercialized Manganese Oxide PLGA NPs

Lastly, the MRI properties of the NPs were measured to evaluate their potential as contrast agents. All NPs displayed a pH-activatable MRI signal as shown in Supporting Figure S12. When the NPs were intact, their relaxivity (*r*_1_) was below 0.7 mM^–1^·s^–1^, representing the “OFF” state as mentioned previously. On the other hand, when the NPs were digested under an acidic environment, the *r*_1_ increased to between ∼3 mM^–1^·s^–1^ and ∼13 mM^–1^·s^–1^, showing the contrast switch to the “ON” state.

**Figure 8:**
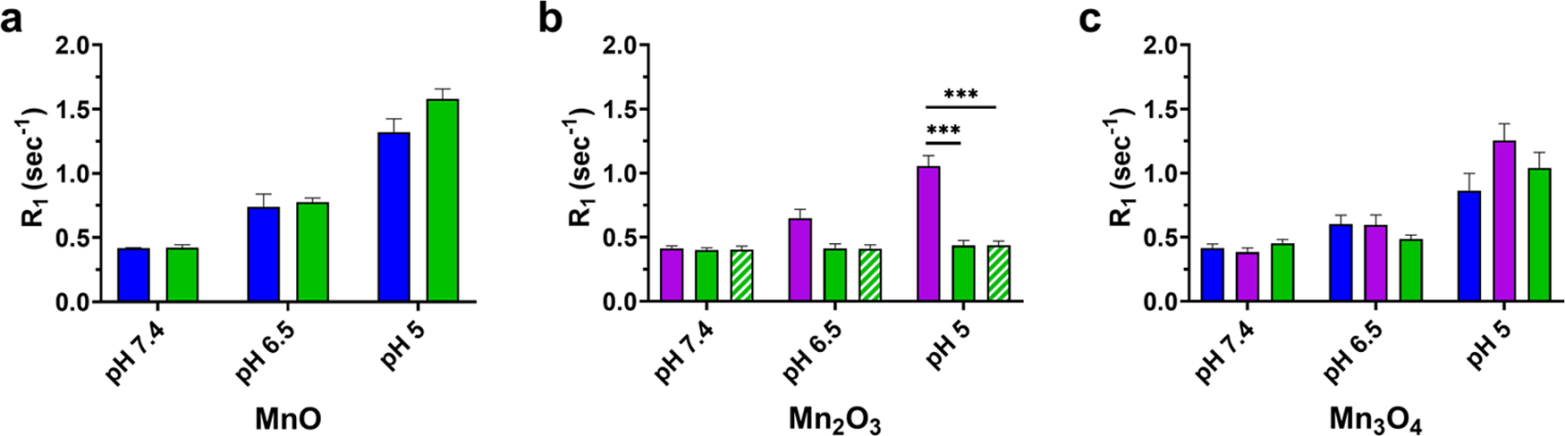
MRI *R*_1_ values of the supernatant collected 1 h after PLGA-encapsulated (a) MnO, (b) Mn_2_O_3_, and (c) Mn_3_O_4_ NP incubation at pH 7.4, pH 6.5, and pH 5. In-house nanocrystals are shown in blue, US Nano’s nanocrystals are displayed in purple, and Nanoshel’s nanocrystals are shown in green (striped bar represents Mn_2_O_3_-80 nm). Note that MnO NPs produced the greatest *R*_1_ at pH 5, followed by Mn_3_O_4_ NPs. Average *R*_1_ values are plotted with standard error of the mean; statistical comparison was performed using two-way ANOVA with Holm–Šídák correction. *P* values are reported as * ≤0.05, *** ≤0.005.

As shown in Figure 8, longitudinal relaxation rates (*R*_1_) were comparable to the controlled release. Both In-house and Nanoshel’s PLGA-encapsulated MnO NPs had the highest *R*_1_ after 1 h at pH 5 with 1.3 and 1.6 s^–1^, respectively. An increase in *R*_1_ indicates that the NPs are more effective contrast agents and are producing bright signal on the MRI scan (Supporting Figure S13). In the case of PLGA Mn_2_O_3_ NPs, US Nano’s NP formulation presented a significantly higher *R*_1_ than both of Nanoshel’s NP formulations at pH 5 due to its increased release of Mn^2+^ (1.1 vs 0.4 s^–1^, respectively); however, the MRI signal remained lower compared to MnO. All PLGA Mn_3_O_4_ NPs had comparable *R*_1_ values (0.86 vs 1.25 vs 1.04 s^–1^, for In-house, US Nano, and Nanoshel, respectively), where US Nano’s Mn_3_O_4_ NPs had better MRI properties than what was synthesized In-house.

Due to their brighter MRI signal, both MnO-based NPs would be effective contrast agents; however, NP size has a significant effect on accumulation within the body. Nanoshel’s PLGA-encapsulated MnO NP size was very large (618 nm) compared to our In-house PLGA MnO NPs (183 nm). Therefore, the ability of these commercially available manganese oxide NPs to reach the tumor is of concern since they significantly fall outside the desired NP size range of 50–200 nm; biodistribution in other organs may also be impacted. Another option could be to use US Nano’s or Nanoshel’s Mn_3_O_4_ since the *R*_1_ values were the closest to the MnO-based NPs (1.25 and 1.04 s^–1^). Out of both options, however, US Nano’s large micron-sized rodlike structures could lead to vessel occlusion in vivo.

## Conclusions

Our results confirm that purchased manganese oxide nanomaterials often do not meet advertised specifications, which can negatively impact experimental applications that depend on the nanomaterial size, morphology, chemistry, dissolution profile, and MRI properties. US Nano’s formulations contained several impurities that affected the release of Mn ions and two distinct size populations including large, rodlike structures that could promote vessel occlusion in vivo. Although Nanoshel’s formulations contained minimal to no impurities, some of their nanocrystals did not adhere to the specified sizes and their MnO- and Mn_2_O_3_-encapsulated NPs displayed large hydrodynamic diameters (>600 nm) that prevent translation to in vivo studies. In contrast, both In-house MnO and Mn_3_O_4_ NPs displayed smaller homogeneous sizes suitable for further preclinical evaluation. In terms of MRI contrast, MnO NPs produced the brightest signal, followed by Mn_3_O_4_ NPs. Mn_2_O_3_ NPs did not dissolve rapidly and resulted in minimal MRI signals and are not recommended for further study as MRI contrast agents. When combining the MRI signal with the hydrodynamic size of all NPs, it was found that In-house MnO NPs were the top contrast agent with Nanoshel’s Mn_3_O_4_ NPs as a close second. To that effect, Nanoshel’s Mn_3_O_4_ formulation will need to be filtered prior to use to remove the possible micron-sized particle populations that were observed with SEM. Our findings highlight the need for researchers to refrain from using purchased nanomaterials without first confirming desired physical, chemical, and magnetic properties—their experimental success may depend on it. In the case of undesired characteristics from commercialized formulations, NP synthesis In-house is a preferred and viable option.

## Methods

### Materials

Manganese(II) acetylacetonate (Mn(II) (AcAc)) (technical grade, ≥97%), oleylamine (technical grade, 70%), and poly(vinyl alcohol) (PVA) were purchased from Sigma-Aldrich. Dibenzyl ether (≥99%, Acros Organics), hexane (≥98.5%, Macron Fine Chemicals), dichloromethane (99.5% stabilized ACS, BDH Chemicals), Dulbecco’s phosphate-buffered saline (PBS), sodium citrate dihydrate (BDH Chemicals), agarose, and citric acid were purchased from VWR Chemicals LLC. Carboxylic acid-terminated, 50:50 poly(d,l-lactide-*co*-glycolide) (PLGA) (inherent viscosity: 0.15–0.25 dL/g) was obtained from LACTEL Absorbable Polymers. Hydrochloric acid (HCl) TraceMetal Grade was acquired from Fisher Scientific. Ethanol (Decon Laboratories, Inc.) was obtained internally from West Virginia University’s Environmental Health and Safety Services. Commercialized Mn_2_O_3_ and Mn_3_O_4_ were purchased from US Research Nanomaterials (US Nano), and commercialized MnO, Mn_2_O_3_-30 nm, Mn_2_O_3_-80 nm, and Mn_3_O_4_ were purchased from Nanoshel. Note, that all experiments and subsequent analyses were performed blindly whenever possible to establish an unbiased approach.

### Synthesis of In-House MnO and Mn_3_O_4_ Nanocrystals

All MnO and Mn_3_O_4_ nanocrystal synthesis steps were performed under a chemical fume hood. Based on previously established methods,^36,37,46^ MnO and Mn_3_O_4_ nanocrystals were produced via thermal decomposition of Mn(II) acetylacetonate (AcAc) with oleylamine and dibenzyl ether.

To synthesize In-house MnO, ∼1.51 g of Mn(II) AcAc was dissolved in 40 mL of oleylamine and 20 mL of dibenzyl ether. The mixture was then heated from room temperature to 60 °C for over 30 min under a constant flow of inert N_2_ gas to ensure the removal of all oxygen to obtain the desired product: MnO nanocrystals. Then, the temperature was raised to 300 °C, at a ramp rate of 20 °C/min, and kept at 300 °C for 30 min.

For In-house Mn_3_O_4_ nanocrystals, the synthesis process was similar, except Mn(II) AcAc was dissolved in 57 mL of oleylamine and 24 mL of dibenzyl ether, heated to 150 °C for 3 h, then rapidly heated to 250 °C with a 10 °C/min ramp, and kept there for 9 h.

For both synthesis techniques, nanocrystals were collected and washed three times with hexane and ethanol at 17,400*g* for 10 min at 10 °C. At the end of the third centrifugation cycle, the MnO and Mn_3_O_4_ nanocrystals were resuspended in hexane and left to dry overnight in a fume hood. After drying overnight, the nanocrystals were baked in a 100 °C oven for 24 h.

### Synthesis of In-House and Commercialized PLGA MnO, Mn_2_O_3_, and Mn_3_O_4_ NPs

In-house and commercialized MnO, Mn_2_O_3_, and Mn_3_O_4_ nanocrystals were encapsulated in PLGA using an oil-in-water emulsion solvent evaporation method as previously described.^36,46^ Each nanocrystal type—In-house MnO and Mn_3_O_4_; US Nano’s Mn_2_O_3_ and Mn_3_O_4_; and Nanoshel’s MnO, Mn_2_O_3_-30 nm, Mn_2_O_3_-80 nm, and Mn_3_O_4_—was encapsulated within PLGA in triplicate. For each replicate, approximately 100 mg of PLGA was dissolved in 2 mL of dichloromethane (DCM). After dissolution, 50 mg of each nanocrystal was added to the polymer/solvent mixture for 8 total samples. The polymer–nanocrystal combination was then bath-sonicated and added dropwise to a 10% aqueous w/v solution of PVA as it was vortexed at high speed. The new mixture was vortexed for 10 s and then sonicated using an ultrasonic processor. Each ultrasonic pulse was applied for 15 s, followed by a 5 s break, repeated three times to create a single emulsion. The emulsion was then poured immediately into an aqueous 0.3% w/v PVA solution. The NP emulsion was stirred for 3 h to facilitate DCM solvent evaporation. Following the evaporation of the DCM, NPs were washed three times with deionized water at 17,400*g* for 10 min at 10 °C. NPs were frozen at −80 °C and subsequently lyophilized.

### X-ray Diffraction (XRD)

A Panalytical X’Pert Pro X-ray diffractometer equipped with a Cu Kα X-ray source operating at 45 kV and 40 mA in the Bragg–Brentano geometry was used to obtain the XRD patterns of bare MnO, Mn_2_O_3_, and Mn_3_O_4_ nanocrystals. A one-dimensional (1D) silicon strip X-ray detector was used to capture spectra throughout a 2θ range of 10 to 90° with a step size of 0.033°. The collected XRD patterns were analyzed using X’Pert HighScore Plus software. The software compared the calculated XRD spectra of the In-house and commercialized nanocrystals against known MnO, Mn_2_O_3_, and Mn_3_O_4_ XRD spectra; the software also searched the XRD database to identify the unknown peaks corresponding to impurities present within commercialized samples.

### Electron Microscopy

Before encapsulation, In-house and commercialized nanocrystals were prepared for transmission electron microscopy (TEM) following previously described methods.^36,37,46^ The particles were imaged using a JEOL JEM-2100 transmission electron microscope at 200 kV. The diameters for the nanocrystals were acquired using ImageJ software.

In-house and commercialized nanocrystals were characterized using scanning electron microscopy (SEM) for chemical composition with a Hitachi SEM S4700 plus energy-dispersive X-ray spectrophotometer (EDS) using the EDAX Team EDS System operated at 15 kV. After encapsulation in PLGA, images of NPs were taken with the Hitachi SEM S4700 operated at 5 kV to evaluate NP morphology.

### Dynamic Light Scattering (DLS)

Hydrodynamic size distributions for the In-house and commercialized NPs suspended in deionized water were measured for each sample using a Malvern Zetasizer Nano ZS (Malvern Instruments). Note that, for NP populations that were polydisperse, the data processing tool “multiple narrow modes” was used.

### Fourier Transform Infrared (FTIR) Spectroscopy

FTIR spectra for all nanocrystals and NPs were obtained using a DIGILAB FTS 7000 FTIR spectrometer equipped with a GladiATR attenuated total reflectance module from PIKE Technologies.

### Thermogravimetric Analysis (TGA)

All nanocrystals were subjected to TGA using an SDT 650 instrument (TA instruments). Briefly, samples were loaded in a chamber, and after sample loading, the chamber was flushed with N_2_ gas for 2 h to establish inert conditions. Then, samples were heated to 105 °C with a 10 °C/min temperature ramp and held there for 1 h to remove any excess water. After removal of excess water, samples were returned to 50 °C and held there for 1 h. Thereafter, data collection for temperature, heat flow, and weight loss was turned on and the temperature was raised to 800 °C with a 5 °C/min temperature ramp. For In-house samples, the weight loss due to oleylamine capping (∼350 °C) was determined for both MnO and Mn_3_O_4_ nanocrystals. Due to the unknown synthesis methods and capping agents used, only nonspecific weight loss was determined for all samples from US Nano and Nanoshel.

### Inductively Coupled Plasma–Optical Emission Spectrometry (ICP-OES)

To evaluate the release of Mn^2+^ content from In-house and commercialized NPs, ICP-OES was used on the collected supernatants following the Mn^2+^-controlled release experiment at different pH levels as previously described.^36,37^ Briefly, approximately 10 mg from each NP sample was added to Eppendorf tubes that held 1 mL of PBS pH 7.4 (blood pH), 20 mM citrate buffer pH 6.5 (tumor microenvironment pH), or 20 mM citrate buffer pH 5 (cellular endosome/lysosome pH). The solutions were incubated at physiological temperature (37 °C), followed by a continuous slow rotation of the tubes to ensure that the samples were gently mixed during the entire incubation. Subsequently, at 1, 2, 4, 8, and 24 h, the Eppendorf tubes were centrifuged at 17,400*g* for 10 min, and the supernatants were collected for ICP-OES analysis of released Mn^2+^ content. The pelleted NPs were resuspended in 1 mL of fresh buffer and placed back into a continuous slow spin until the next time point was collected. At the end of collections, the amounts of Mn^2+^ present were measured using an Agilent 720 ICP-OES (1400 watts) with a plasma flow of 15.0 L/min, an auxiliary flow of 1.50 L/min, and a nebulizer flow of 0.75 L/min. The percent Mn^2+^ released at each time point and encapsulation efficiency were calculated using already established equations found in the previous literature.^36^

### Magnetic Resonance Imaging (MRI)

MRI experiments were performed as described previously.^36^ Briefly, supernatants from the eight NP sample types at three different pH conditions were collected after 1 h during the Mn^2+^ release experiment as above. Supernatants were diluted 100-fold and then analyzed for their longitudinal MRI properties in a 1.0 T Bruker ICON MRI. *R*_1_ values were acquired using a RARE sequence with an echo time of 10.68 ms and a repetition time ranging from 25.6 to 12,800 ms. Images were then evaluated with ImageJ, and data were fitted to follow the *R*_1_ longitudinal relaxation equation (eq 6 below) using MATLAB.
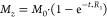
6where *M*_*z*_ is the longitudinal magnetization aligned along the *z*-axis at some time, *t*, and *M*_0_ is the magnetization at equilibrium.

Additionally, intact NPs suspended in 0.5% agarose- and HCl-digested NPs were imaged at different concentrations of Mn following the same protocol above. Data were then plotted and fitted to follow eq 7 to find the longitudinal relaxivity (*r*_1_) properties of the NPs.
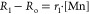
7where *R*_o_ is the longitudinal relaxation rate when no Mn is present and [Mn] is the concentration of manganese in mM.

### Statistical Analysis

All statistical analysis was performed in GraphPad Prism V 9.4.1 by applying ANOVA with Holm–Šídák correction. *P* values <0.05 were considered significant.

## Data Availability

All data generated or analyzed during this study are included in this published article [and its Supporting Information files].

## Supporting Information Available

XRD percent composition for all In-house and commercialized nanocrystals via X-Pert HighScore (Table S1); XRD spectra of US Nano’s Mn_2_O_3_ and Mn_3_O_4_ nanocrystals to highlight impurities (Figure S1); EDS spectra of all In-house and commercialized nanocrystal formulations (Figure S2); EDS mapping of US Nano’s Mn_2_O_3_ and Mn_3_O_4_ nanocrystals to further analyze impurities (Figure S3); FTIR spectra for all In-house and commercialized nanocrystal formulations (Figure S4); TGA for all In-house and commercialized nanocrystal formulations (Figure S5); SEM images of PLGA-encapsulated In-house and commercialized manganese oxide NPs (Figure S6); FTIR spectra for batch #1 of PLGA-encapsulated In-house and commercialized manganese oxide NPs (Figure S7); average hydrodynamic size, encapsulation efficiency, and yield for NEMO particles (Table S2); DLS size distribution for batches #1–3 of PLGA-encapsulated In-house and commercialized manganese oxide NPs (Figure S8–S10); cumulative release of Mn^2+^ from PLGA-encapsulated In-house and commercialized manganese oxide NPs (Figure S11); longitudinal relaxivity *r*_1_ properties of PLGA-encapsulated In-house and commercialized manganese oxide NPs (Figure S12); and MRI properties of Mn^2+^ supernatants collected from dissolving PLGA-encapsulated In-house and commercialized manganese oxide NPs (Figure 13) (PDF)

## References

[ref1] de Haën C. Conception of the first magnetic resonance imaging contrast agents: a brief history. Top. Magn. Reson. Imaging 2001, 12, 221–230. 10.1097/00002142-200108000-00002. 11687712

[ref2] Rogosnitzky M.; Branch S. Gadolinium-based contrast agent toxicity: a review of known and proposed mechanisms. BioMetals 2016, 29, 365–376. 10.1007/s10534-016-9931-7. 27053146 PMC4879157

[ref3] Wahsner J.; Gale E. M.; Rodríguez-Rodríguez A.; Caravan P. Chemistry of MRI Contrast Agents: Current Challenges and New Frontiers. Chem. Rev. 2019, 119, 957–1057. 10.1021/acs.chemrev.8b00363. 30350585 PMC6516866

[ref4] Ferris N.; Goergen S. Gadolinium Contrast Medium (MRI Contrast Agents), 2017. https://www.insideradiology.com.au/gadolinium-contrast-medium/.

[ref5] Zhou Z.; Lu Z. R. Gadolinium-based contrast agents for magnetic resonance cancer imaging. Wiley Interdiscip. Rev.: Nanomed. Nanobiotechnol. 2013, 5, 1–18. 10.1002/wnan.1198. 23047730 PMC3552562

[ref6] Guo B. J.; Yang Z. L.; Zhang L. J. Gadolinium Deposition in Brain: Current Scientific Evidence and Future Perspectives. Front. Mol. Neurosci. 2018, 11, 335 10.3389/fnmol.2018.00335. 30294259 PMC6158336

[ref7] Leyba K.; Wagner B. Gadolinium-based contrast agents: why nephrologists need to be concerned. Curr. Opin. Nephrol. Hypertens. 2019, 28, 154–162. 10.1097/MNH.0000000000000475. 30531473 PMC6416778

[ref8] Ranga A.; Agarwal Y.; Garg K. J. Gadolinium based contrast agents in current practice: Risks of accumulation and toxicity in patients with normal renal function. Indian J. Radiol Imaging 2017, 27, 141–147. 10.4103/0971-3026.209212. 28744073 PMC5510310

[ref9] Wei H.; Bruns O. T.; Kaul M. G.; Hansen E. C.; Barch M.; Wiśniowska A.; Chen O.; Chen Y.; Li N.; Okada S.; Cordero J. M.; Heine M.; Farrar C. T.; Montana D. M.; Adam G.; Ittrich H.; Jasanoff A.; Nielsen P.; Bawendi M. G. Exceedingly small iron oxide nanoparticles as positive MRI contrast agents. Proc. Natl. Acad. Sci. U.S.A. 2017, 114, 2325–2330. 10.1073/pnas.1620145114. 28193901 PMC5338531

[ref10] Cai X.; Zhu Q.; Zeng Y.; Zeng Q.; Chen X.; Zhan Y. Manganese Oxide Nanoparticles As MRI Contrast Agents In Tumor Multimodal Imaging And Therapy. Int. J. Nanomed. 2019, 14, 8321–8344. 10.2147/IJN.S218085. PMC6814316 31695370

[ref11] Fei J.; Sun L.; Zhou C.; Ling H.; Yan F.; Zhong X.; Lu Y.; Shi J.; Huang J.; Liu Z. Tuning the Synthesis of Manganese Oxides Nanoparticles for Efficient Oxidation of Benzyl Alcohol. Nanoscale Res. Lett. 2017, 12, 23 10.1186/s11671-016-1777-y. 28063142 PMC5218959

[ref12] Kuo C.-H.; Mosa I. M.; Poyraz A. S.; Biswas S.; El-Sawy A. M.; Song W.; Luo Z.; Chen S.-Y.; Rusling J. F.; He J.; Suib S. L. Robust Mesoporous Manganese Oxide Catalysts for Water Oxidation. ACS Catal. 2015, 5, 1693–1699. 10.1021/cs501739e.

[ref13] Kim S. C.; Shim W. G. Catalytic combustion of VOCs over a series of manganese oxide catalysts. Appl. Catal., B 2010, 98, 180–185. 10.1016/j.apcatb.2010.05.027.

[ref14] Farzana R.; Rajarao R.; Hassan K.; Behera P. R.; Sahajwalla V. Thermal nanosizing: Novel route to synthesize manganese oxide and zinc oxide nanoparticles simultaneously from spent Zn–C battery. J. Cleaner Prod. 2018, 196, 478–488. 10.1016/j.jclepro.2018.06.055.

[ref15] Liu X.; Chen C.; Zhao Y.; Jia B. A Review on the Synthesis of Manganese Oxide Nanomaterials and Their Applications on Lithium-Ion Batteries. J. Nanomater. 2013, 2013, 1–7. 10.1155/2013/736375.

[ref16] Zhang Y.; Steiner J. D.; Uzodinma J.; Walsh J.; Zydlewski B.; Lin F.; Chen Y.; Tang J.; Pradhan N.; Dai Q. Thermally synthesized MnO nanoparticles for magnetic properties and lithium batteries. Mater. Res. Express 2018, 6, 025015 10.1088/2053-1591/aaecc8.

[ref17] Ahmed S.; Ahmad Z.; Kumar A.; Rafiq M.; Vashistha V. K.; Ashiq M. N.; Kumar A. Effective removal of methylene blue using nanoscale manganese oxide rods and spheres derived from different precursors of manganese. J. Phys. Chem. Solids 2021, 155, 110121 10.1016/j.jpcs.2021.110121.

[ref18] Chen H.; He J. Facile Synthesis of Monodisperse Manganese Oxide Nanostructures and Their Application in Water Treatment. J. Phys. Chem. C 2008, 112, 17540–17545. 10.1021/jp806160g.

[ref19] George J. M.; Antony A.; Mathew B. Metal oxide nanoparticles in electrochemical sensing and biosensing: a review. Mikrochim. Acta 2018, 185, 358 10.1007/s00604-018-2894-3. 29974265

[ref20] Vukojević V.; Djurdjić S.; Ognjanović M.; Fabián M.; Samphao A.; Kalcher K.; Stanković D. M. Enzymatic glucose biosensor based on manganese dioxide nanoparticles decorated on graphene nanoribbons. J. Electroanal. Chem. 2018, 823, 610–616. 10.1016/j.jelechem.2018.07.013.

[ref21] Ding B.; Zheng P.; Ma P.; Lin J. Manganese Oxide Nanomaterials: Synthesis, Properties, and Theranostic Applications. Adv. Mater. 2020, 32, 1905823 10.1002/adma.201905823. 31990409

[ref22] Felton C.; Karmakar A.; Gartia Y.; Ramidi P.; Biris A. S.; Ghosh A. Magnetic nanoparticles as contrast agents in biomedical imaging: recent advances in iron- and manganese-based magnetic nanoparticles. Drug Metab. Rev. 2014, 46, 142–154. 10.3109/03602532.2013.876429. 24754519

[ref23] Hsu B. Y. W.; Kirby G.; Tan A.; Seifalian A. M.; Li X.; Wang J. Relaxivity and toxicological properties of manganese oxide nanoparticles for MRI applications. RSC Adv. 2016, 6, 45462–45474. 10.1039/C6RA04421B. 31156805 PMC6542684

[ref24] Estelrich J.; Sánchez-Martín M. J.; Busquets M. A. Nanoparticles in magnetic resonance imaging: from simple to dual contrast agents. Int. J. Nanomed. 2015, 10, 1727–1741. 10.2147/IJN.S76501. PMC4358688 25834422

[ref25] Anderson D.; Anderson T.; Fahmi F. Advances in Applications of Metal Oxide Nanomaterials as Imaging Contrast Agents. Phys. Status Solidi A 2019, 216, 1801008 10.1002/pssa.201801008.

[ref26] Li J.; Wu C.; Hou P.; Zhang M.; Xu K. One-pot preparation of hydrophilic manganese oxide nanoparticles as T(1) nano-contrast agent for molecular magnetic resonance imaging of renal carcinoma in vitro and in vivo. Biosens. Bioelectron. 2018, 102, 1–8. 10.1016/j.bios.2017.10.047. 29101783

[ref27] Bennewitz M. F.; Lobo T. L.; Nkansah M. K.; Ulas G.; Brudvig G. W.; Shapiro E. M. Biocompatible and pH-sensitive PLGA encapsulated MnO nanocrystals for molecular and cellular MRI. ACS Nano 2011, 5, 3438–3446. 10.1021/nn1019779. 21495676 PMC3102302

[ref28] Millet I.; Pages E.; Hoa D.; Merigeaud S.; Curros Doyon F.; Prat X.; Taourel P. Pearls and pitfalls in breast MRI. Br. J. Radiol. 2012, 85, 197–207. 10.1259/bjr/47213729. 22128131 PMC3473994

[ref29] Shin Y.; Sohn Y. M.; Seo M.; Han K. False-negative results of breast MR computer-aided evaluation in patients with breast cancer: correlation with clinicopathologic and radiologic factors. Clin. Imaging 2016, 40, 1086–1091. 10.1016/j.clinimag.2016.06.010. 27421083

[ref30] Giess C. S.; Yeh E. D.; Raza S.; Birdwell R. L. Background parenchymal enhancement at breast MR imaging: normal patterns, diagnostic challenges, and potential for false-positive and false-negative interpretation. RadioGraphics 2014, 34, 234–247. 10.1148/rg.341135034. 24428293

[ref31] Shimauchi A.; Jansen S. A.; Abe H.; Jaskowiak N.; Schmidt R. A.; Newstead G. M. Breast cancers not detected at MRI: review of false-negative lesions. Am. J. Roentgenol. 2010, 194, 1674–1679. 10.2214/AJR.09.3568. 20489112

[ref32] Wang X.; Niu D.; Wu Q.; Bao S.; Su T.; Liu X.; Zhang S.; Wang Q. Iron oxide/manganese oxide co-loaded hybrid nanogels as pH-responsive magnetic resonance contrast agents. Biomaterials 2015, 53, 349–357. 10.1016/j.biomaterials.2015.02.101. 25890733

[ref33] Rosen J. E.; Chan L.; Shieh D. B.; Gu F. X. Iron oxide nanoparticles for targeted cancer imaging and diagnostics. Nanomedicine 2012, 8, 275–290. 10.1016/j.nano.2011.08.017. 21930108

[ref34] Moore A.; Medarova Z.; Potthast A.; Dai G. In vivo targeting of underglycosylated MUC-1 tumor antigen using a multimodal imaging probe. Cancer Res. 2004, 64, 1821–1827. 10.1158/0008-5472.CAN-03-3230. 14996745

[ref35] Delehanty J. B.; Boeneman K.; Bradburne C. E.; Robertson K.; Bongard J. E.; Medintz I. L. Peptides for specific intracellular delivery and targeting of nanoparticles: implications for developing nanoparticle-mediated drug delivery. Ther. Delivery 2010, 1, 411–433. 10.4155/tde.10.27. 22816144

[ref36] de la Torre C. M.; Grossman J. H.; Bobko A. A.; Bennewitz M. F. Tuning the size and composition of manganese oxide nanoparticles through varying temperature ramp and aging time. PLoS One 2020, 15, e0239034 10.1371/journal.pone.0239034. 32946514 PMC7500698

[ref37] Martinez de la Torre C.; Bennewitz M. F. Manganese Oxide Nanoparticle Synthesis by Thermal Decomposition of Manganese(II) Acetylacetonate. J. Visualized Exp. 2020, e61572 10.3791/61572-v. 32628168

[ref38] Xiao J.; Tian X. M.; Yang C.; Liu P.; Luo N. Q.; Liang Y.; Li H. B.; Chen D. H.; Wang C. X.; Li L.; Yang G. W. Ultrahigh relaxivity and safe probes of manganese oxide nanoparticles for in vivo imaging. Sci. Rep. 2013, 3, 3424 10.1038/srep03424. 24305731 PMC4070373

[ref39] Paliwal R.; Babu R. J.; Palakurthi S. Nanomedicine scale-up technologies: feasibilities and challenges. AAPS PharmSciTech 2014, 15, 1527–1534. 10.1208/s12249-014-0177-9. 25047256 PMC4245446

[ref40] Operti M. C.; Bernhardt A.; Grimm S.; Engel A.; Figdor C. G.; Tagit O. PLGA-based nanomedicines manufacturing: Technologies overview and challenges in industrial scale-up. Int. J. Pharm. 2021, 605, 120807 10.1016/j.ijpharm.2021.120807. 34144133

[ref41] Hernández-Giottonini K. Y.; Rodríguez-Córdova R. J.; Gutiérrez-Valenzuela C. A.; Peñuñuri-Miranda O.; Zavala-Rivera P.; Guerrero-Germán P.; Lucero-Acuña A. PLGA nanoparticle preparations by emulsification and nanoprecipitation techniques: effects of formulation parameters. RSC Adv. 2020, 10, 4218–4231. 10.1039/C9RA10857B. 35495261 PMC9049000

[ref42] Raliya R.; Chadha T. S.; Haddad K.; Biswas P. Perspective on Nanoparticle Technology for Biomedical Use. Curr. Pharm. Des. 2016, 22, 2481–2490. 10.2174/1381612822666160307151409. 26951098 PMC4930863

[ref43] Mu Y.; Wu F.; Zhao Q.; Ji R.; Qie Y.; Zhou Y.; Hu Y.; Pang C.; Hristozov D.; Giesy J. P.; Xing B. Predicting toxic potencies of metal oxide nanoparticles by means of nano-QSARs. Nanotoxicology 2016, 10, 1207–1214. 10.1080/17435390.2016.1202352. 27309010

[ref44] Heinlaan M.; Muna M.; Juganson K.; Oriekhova O.; Stoll S.; Kahru A.; Slaveykova V. I. Exposure to sublethal concentrations of Co(3)O(4) and Mn(2)O(3) nanoparticles induced elevated metal body burden in Daphnia magna. Aquat. Toxicol. 2017, 189, 123–133. 10.1016/j.aquatox.2017.06.002. 28623688

[ref45] Razumov I. A.; Zav’yalov E. L.; Troitskii S. Y.; Romashchenko A. V.; Petrovskii D. V.; Kuper K. E.; Moshkin M. P. Selective Cytotoxicity of Manganese Nanoparticles against Human Glioblastoma Cells. Bull. Exp. Biol. Med. 2017, 163, 561–565. 10.1007/s10517-017-3849-0. 28853080

[ref46] Snoderly H. T.; Freshwater K. A.; de la Torre C. M.; Panchal D. M.; Vito J. N.; Bennewitz M. F. PEGylation of Metal Oxide Nanoparticles Modulates Neutrophil Extracellular Trap Formation. Biosensors 2022, 12, 123 10.3390/bios12020123. 35200382 PMC8869785

[ref47] Raja P. M. V.; Barron A. R. An Introduction to Energy Dispersive X-ray Spectroscopy. Chemistry LibreTexts, 2022. https://chem.libretexts.org/Bookshelves/Analytical_Chemistry/Physical_Methods_in_Chemistry_and_Nano_Science_(Barron)/01%3A_Elemental_Analysis/1.12%3A_An_Introduction_to_Energy_Dispersive_X-ray_Spectroscopy.

[ref48] Stojilovic N. Why Can’t We See Hydrogen in X-ray Photoelectron Spectroscopy?. J. Chem. Educ. 2012, 89, 1331–1332. 10.1021/ed300057j.

[ref49] Antoni H.; Xia W.; Masa J.; Schuhmann W.; Muhler M. Tuning the oxidation state of manganese oxide nanoparticles on oxygen- and nitrogen-functionalized carbon nanotubes for the electrocatalytic oxygen evolution reaction. Phys. Chem. Chem. Phys. 2017, 19, 18434–18442. 10.1039/C7CP02717F. 28678247

[ref50] An K.; Park M.; Yu J. H.; Na H. B.; Lee N.; Park J.; Choi S. H.; Song I. C.; Moon W. K.; Hyeon T. Synthesis of Uniformly Sized Manganese Oxide Nanocrystals with Various Sizes and Shapes and Characterization of Their T1 Magnetic Resonance Relaxivity. Eur. J. Inorg. Chem. 2012, 2012, 2148–2155. 10.1002/ejic.201101193.

[ref51] Cheng F.; Shen J.; Ji W.; Tao Z.; Chen J. Selective Synthesis of Manganese Oxide Nanostructures for Electrocatalytic Oxygen Reduction. ACS Appl. Mater. Interfaces 2009, 1, 460–466. 10.1021/am800131v. 20353237

[ref52] Farmer S.; Kennepohl D.; Reusch W.; Reusch W. Interpreting Infrared Spectra. Chemistry LibreTexts, 2023. https://chem.libretexts.org/Bookshelves/Organic_Chemistry/Organic_Chemistry_(Morsch_et_al.)/12%3A_Structure_Determination_-_Mass_Spectrometry_and_Infrared_Spectroscopy/12.07%3A_Interpreting_Infrared_Spectra.

[ref53] Aggarwal P.; Hall J. B.; McLeland C. B.; Dobrovolskaia M. A.; McNeil S. E. Nanoparticle interaction with plasma proteins as it relates to particle biodistribution, biocompatibility and therapeutic efficacy. Adv. Drug Delivery Rev. 2009, 61, 428–437. 10.1016/j.addr.2009.03.009. PMC3683962 19376175

[ref54] Marucco A.; Aldieri E.; Leinardi R.; Bergamaschi E.; Riganti C.; Fenoglio I. Applicability and Limitations in the Characterization of Poly-Dispersed Engineered Nanomaterials in Cell Media by Dynamic Light Scattering (DLS). Materials 2019, 12, 3833 10.3390/ma12233833. 31766412 PMC6926523

[ref55] Stetefeld J.; McKenna S. A.; Patel T. R. Dynamic light scattering: a practical guide and applications in biomedical sciences. Biophys. Rev. 2016, 8, 409–427. 10.1007/s12551-016-0218-6. 28510011 PMC5425802

[ref56] Malvern_Panalytical. Dynamic Light Scattering (DLS—Understanding the Basics, 2013. https://www.azonano.com/article.aspx?ArticleID=3662#.

[ref57] Blanco E.; Shen H.; Ferrari M. Principles of nanoparticle design for overcoming biological barriers to drug delivery. Nat. Biotechnol. 2015, 33, 941–951. 10.1038/nbt.3330. 26348965 PMC4978509

[ref58] Gao Z.; Ma T.; Zhao E.; Docter D.; Yang W.; Stauber R. H.; Gao M. Small is Smarter: Nano MRI Contrast Agents - Advantages and Recent Achievements. Small 2016, 12, 556–576. 10.1002/smll.201502309. 26680328

[ref59] Perry J. L.; Reuter K. G.; Luft J. C.; Pecot C. V.; Zamboni W.; DeSimone J. M. Mediating Passive Tumor Accumulation through Particle Size, Tumor Type, and Location. Nano Lett. 2017, 17, 2879–2886. 10.1021/acs.nanolett.7b00021. 28287740 PMC5708115

[ref60] Tang L.; Yang X.; Yin Q.; Cai K.; Wang H.; Chaudhury I.; Yao C.; Zhou Q.; Kwon M.; Hartman J. A.; Dobrucki I. T.; Dobrucki L. W.; Borst L. B.; Lezmi S.; Helferich W. G.; Ferguson A. L.; Fan T. M.; Cheng J. Investigating the optimal size of anticancer nanomedicine. Proc. Natl. Acad. Sci. U.S.A. 2014, 111, 15344–15349. 10.1073/pnas.1411499111. 25316794 PMC4217425

[ref61] Sarin H. Physiologic upper limits of pore size of different blood capillary types and another perspective on the dual pore theory of microvascular permeability. J. Angiog. Res. 2010, 2, 14 10.1186/2040-2384-2-14. PMC2928191 20701757

[ref62] Yu W.; Liu R.; Zhou Y.; Gao H. Size-Tunable Strategies for a Tumor Targeted Drug Delivery System. ACS Cent. Sci. 2020, 6, 100–116. 10.1021/acscentsci.9b01139. 32123729 PMC7047275

[ref63] Godunov E. B.; Izotov A. D.; Gorichev I. G. Dissolution of Manganese Oxides of Various Compositions in Sulfuric Acid Solutions Studied by Kinetic Methods. Inorg. Mater. 2018, 54, 66–71. 10.1134/S002016851801003X.

[ref64] Rodrigues S.; Shukla A. K.; Munichandraiah N. A cyclic voltammetric study of the kinetics andmechanism of electrodeposition of manganese dioxide. J. Appl. Electrochem. 1998, 28, 1235–1241. 10.1023/A:1003472901760.

[ref65] Bock N. A.; Paiva F. F.; Silva A. C. Fractionated manganese-enhanced MRI. NMR Biomed. 2008, 21, 473–478. 10.1002/nbm.1211. 17944008 PMC4748952

[ref66] Zhan Y.; Zhan W.; Li H.; Xu X.; Cao X.; Zhu S.; Liang J.; Chen X. In Vivo Dual-Modality Fluorescence and Magnetic Resonance Imaging-Guided Lymph Node Mapping with Good Biocompatibility Manganese Oxide Nanoparticles. Molecules 2017, 22, 2208 10.3390/molecules22122208. 29231865 PMC6149721

[ref67] Zheng Y.; Zhang H.; Hu Y.; Bai L.; Xue J. MnO nanoparticles with potential application in magnetic resonance imaging and drug delivery for myocardial infarction. Int. J. Nanomed. 2018, 13, 6177–6188. 10.2147/IJN.S176404. PMC6181115 30323598

