# Supplementary material for: Caveat Emptor: Commercialized Manganese Oxide Nanoparticles Exhibit Unintended Properties
Source: ACS Omega. 2023 May 18;8(21):18799–810. doi: 10.1021/acsomega.3c00892 (PMC10233837; doi:10.1021/acsomega.3c00892)
Supplement: Supplementary file 1 — ao3c00892_si_001.pdf [file ao3c00892_si_001.pdf]

Supporting Information

Caveat Emptor: Commercialized Manganese Oxide Nanoparticles Exhibit Unintended Properties

Celia Martinez de la Torre[1†], Kasey A. Freshwater[1†], Mara A. Looney-Sanders[1], Qiang Wang[2], and Margaret F. Bennewitz[1*]

[1] Department of Chemical and Biomedical Engineering, Benjamin M. Statler College of Engineering and Mineral Resources, West Virginia University, Morgantown, WV 26506, USA

[2] Shared Research Facilities, West Virginia University, Morgantown, WV 26506, USA

[†]C.M.T. and K.A.F. contributed equally to this work and are co-first authors.

*Corresponding author:

Margaret F. Bennewitz, PhD
Assistant Professor
Department of Chemical and Biomedical Engineering
West Virginia University
Morgantown, WV 26506
E-mail: margaret.bennewitz@mail.wvu.edu

Table S1. XRD percent composition for all nanocrystals via X-Pert HighScore.

Nanocrystal Type		Composition (%)					
		Marketed Purity	MnO	Mn_2O_3	Mn_3O_4	MnO_2	Other impurities
**In-house**	MnO	N/A	**100**	-	-	-	-
	Mn_3O_4	N/A	-	-	**100**	-	-
**US Nano**	Mn_2O_3	>99.2	-	**0**	55.7	-	44.3*
	Mn_3O_4	>99.95	-	-	**69.1**	-	30.9ǂ
**Nanoshel**	MnO	>99.9	**100**	-	-	-	-
	Mn_2O_3-30nm	>99.9	-	**98.7**	-	1.3	-
	Mn_2O_3-80nm	>99.9	-	**98.6**	-	1.4	-
	Mn_3O_4	>99.9	-	2.1	**97.9**	**-**	**-**

The corresponding percent composition for an intended formulation is shown in bold.

*Other impurities for US Nano's Mn_2O_3 included: 39.3% $Na_{0.58}Mn_{1.42}Mn_{0.58}O_4{\bullet}(H_2O)_{1.38}$, 3.6% $CaMn_7O_{12}$, and 1.4% SiO_2.

ǂOther impurities for US Nano's Mn_3O_4 included: 30.9% $HMnO_2$.

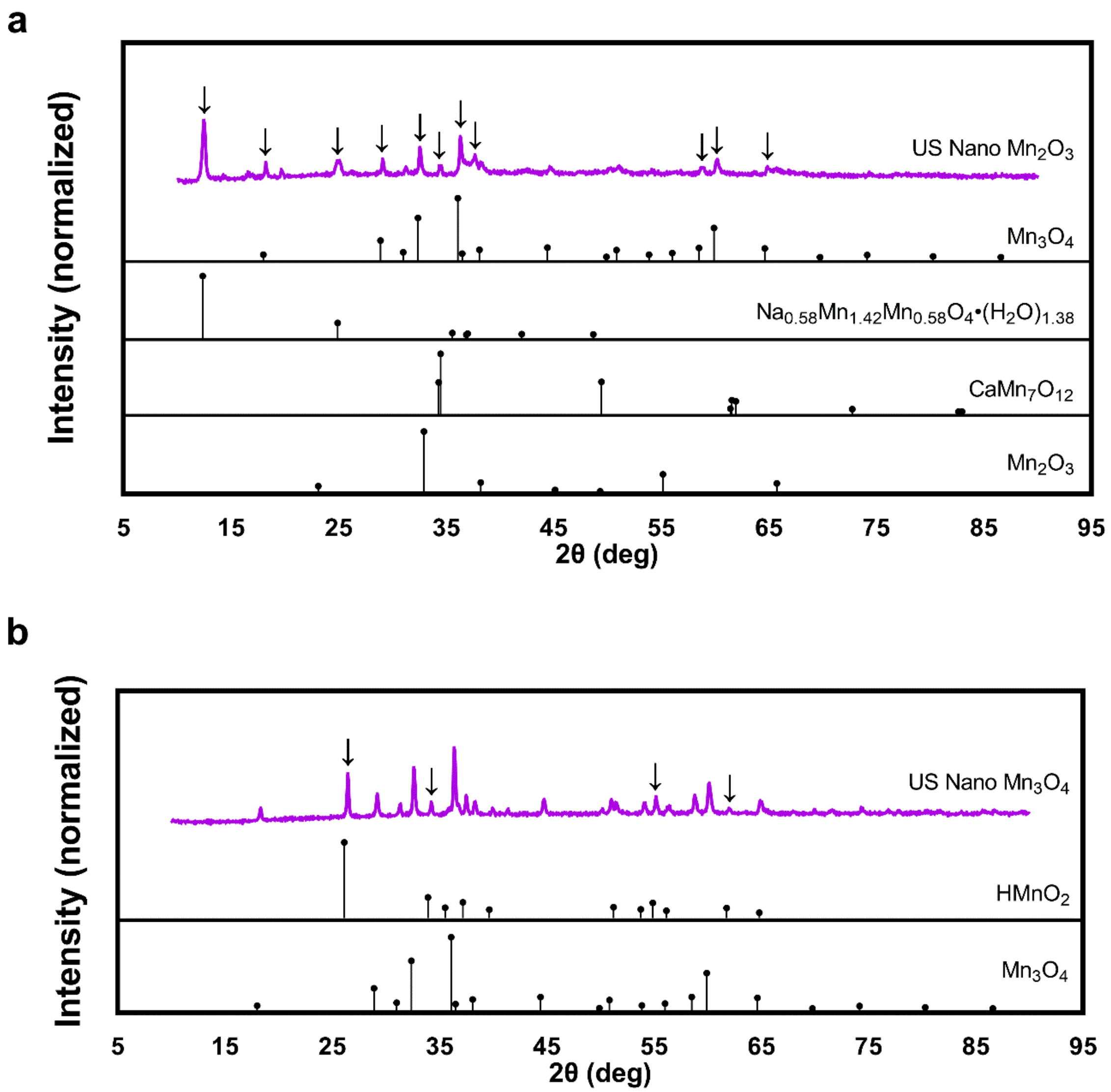

Figure S1. XRD spectra of US Nano's (a) Mn_2O_3 and (b) Mn_3O_4 nanocrystals highlight impurities. The corresponding characteristic peaks are shown in black for other matching crystal structures and the intended crystal structure. Peaks corresponding to the possible impurities are marked by arrows. Note that US Nano's Mn_2O_3 nanocrystals contained very minimal additional SiO_2 contamination (1.4%), which is not shown here; this sample also showed no peaks in common with the standard diffraction XRD spectra for Mn_2O_3, the intended compound.

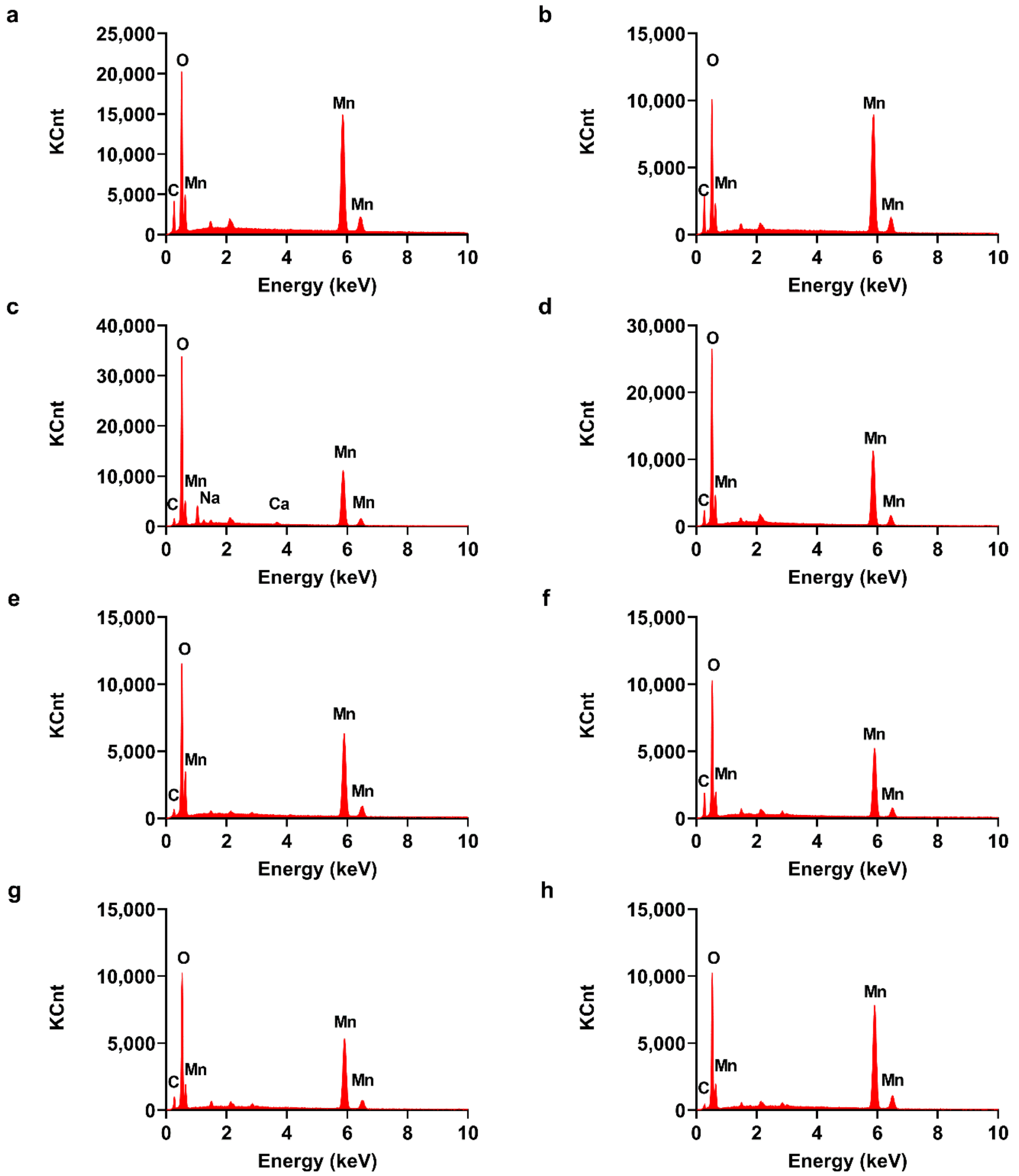

Figure S2. EDS spectra of In-house (a) MnO and (b) Mn_3O_4, US Nano's (c) Mn_2O_3 and (d) Mn_3O_4, and Nanoshel's (e) MnO, (f) Mn_2O_3-30 nm, (g) Mn_2O_3-80 nm, and (h) Mn_3O_4 nanocrystals**.** All samples present manganese, oxygen, and carbon, except for (c) US Nano's Mn_2O_3, which shows sodium and calcium impurities.

a

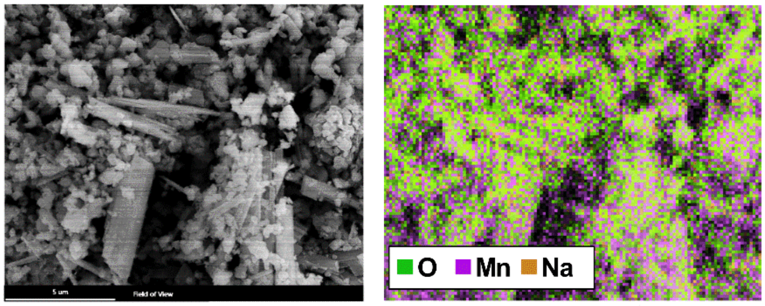

Element	Atomic %
O	50.04
Mn	36.58
Na	13.38

b

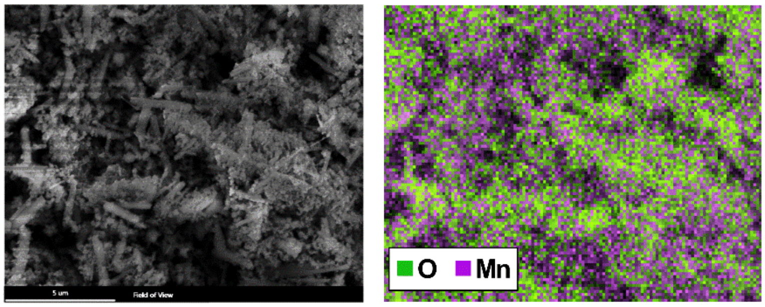

Element	Atomic %
O	48.70
Mn	51.30

Figure S3. EDS mapping of US Nano's (a) Mn_2O_3 and (b) Mn_3O_4 nanocrystals to further analyze the impurities present. On the left, the SEM images show the metal oxide nanocrystals. The middle images display the elements detected during the mapping scans (green for oxygen, purple for manganese, and orange for sodium) over the same area as the SEM images. The right tables highlight the atomic composition from each sample. Note the sodium impurity detected in US Nano's Mn_2O_3 was spread uniformly over the nanocrystals.

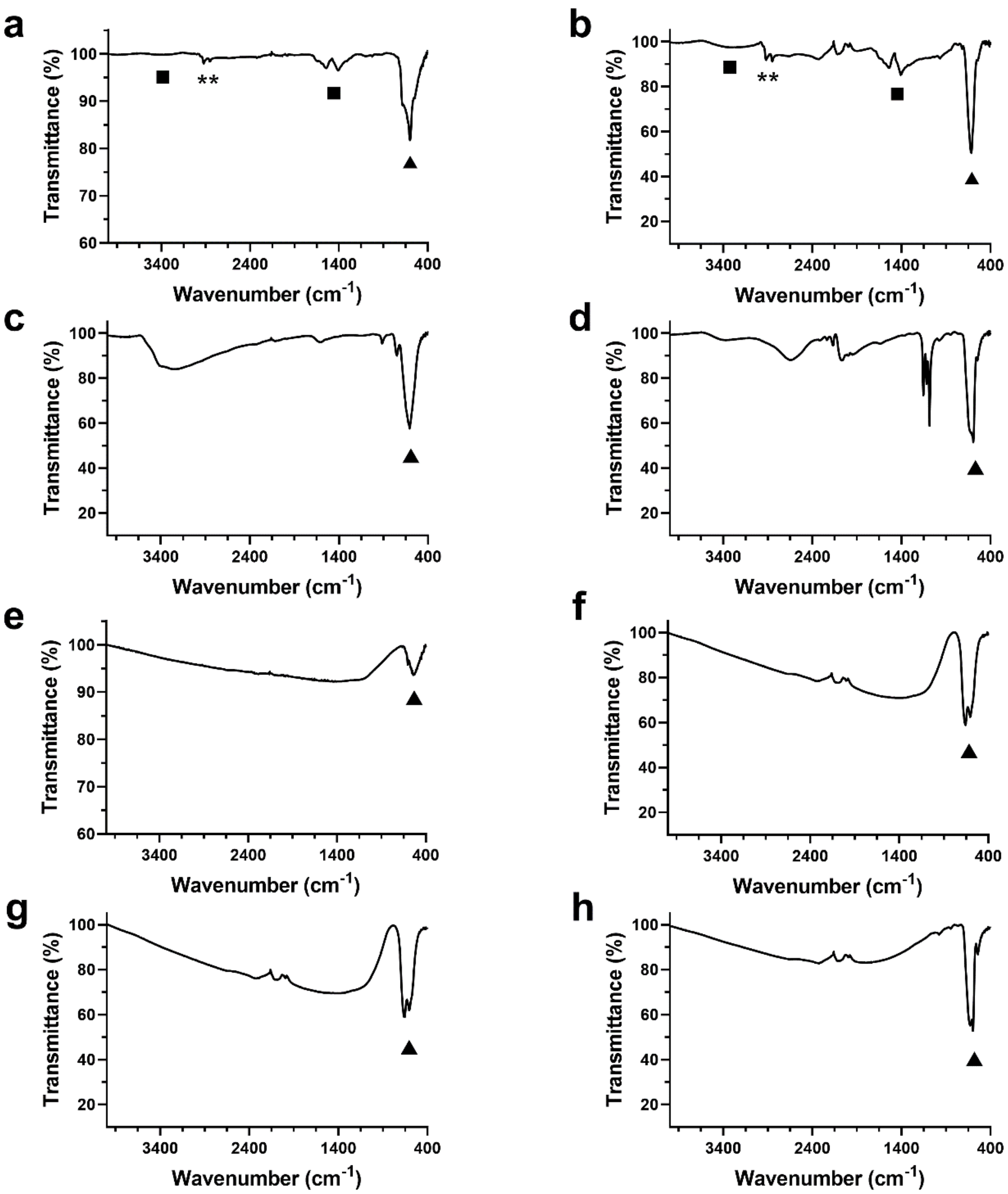

Figure S4. FTIR spectra for In-house (a) MnO and (b) Mn_3O_4, US Nano's (c) Mn_2O_3 and (d) Mn_3O_4, and Nanoshel's (e) MnO, (f) Mn_2O_3-30 nm, (g) Mn_2O_3-80 nm, and (h) Mn_3O_4 nanocrystals. Representative peaks for oleyl groups (∗), amine groups (■), and manganese oxide (▲) are shown for In-house nanocrystals.[1-4] Due to unknown synthesis techniques, only representative peaks for manganese oxide (▲) are shown for US Nano and Nanoshel.

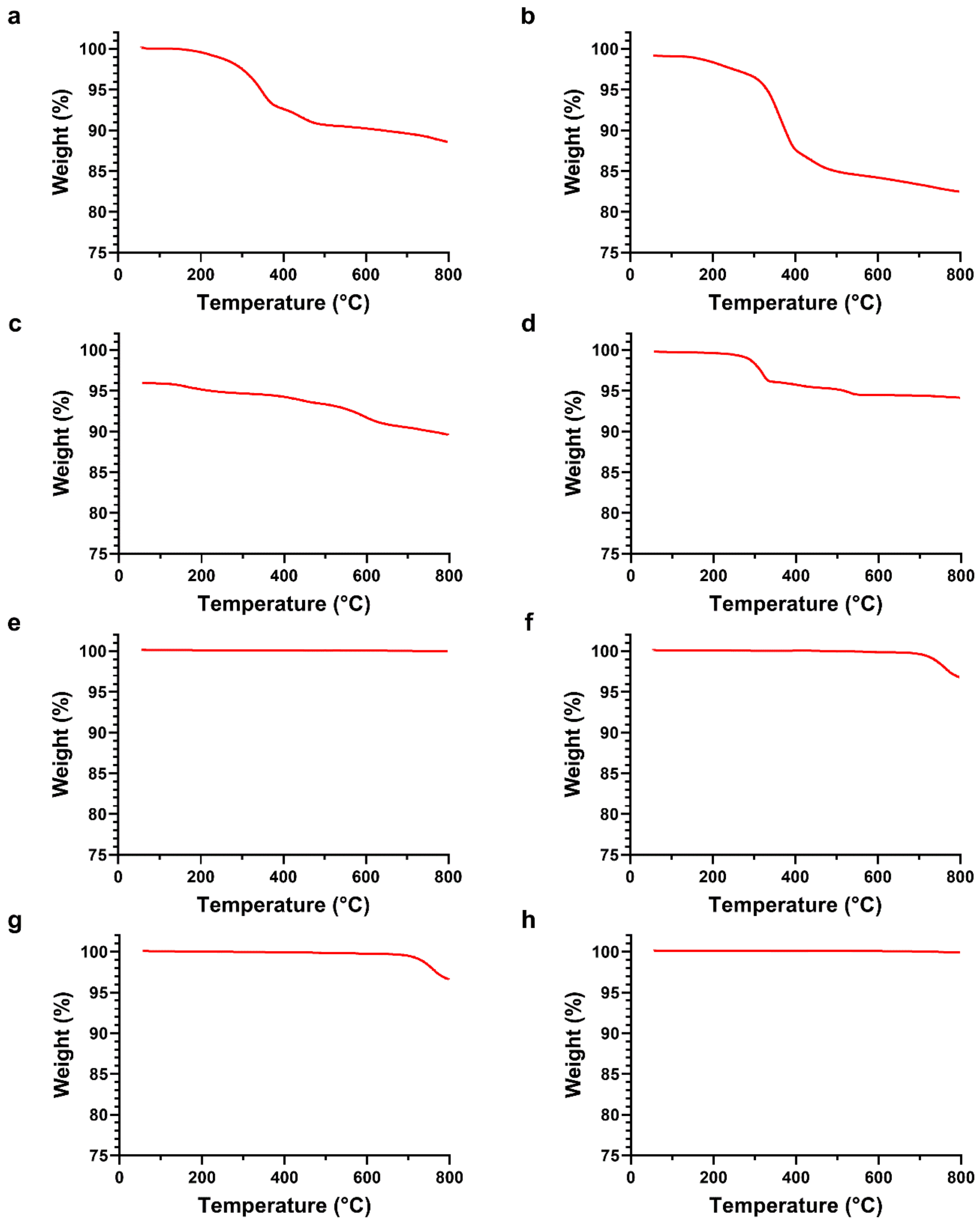

Figure S5. ThermoGravimetric Analysis (TGA) for In-house (a) MnO and (b) Mn_3O_4, US Nano's (c) Mn_2O_3 and (d) Mn_3O_4, and Nanoshel's (e) MnO, (f) Mn_2O_3-30 nm, (g) Mn_2O_3-80 nm, and (h) Mn_3O_4 nanocrystals. For In-house nanocrystals, the decrease in weight at ~350°C was due to the oleylamine coating.

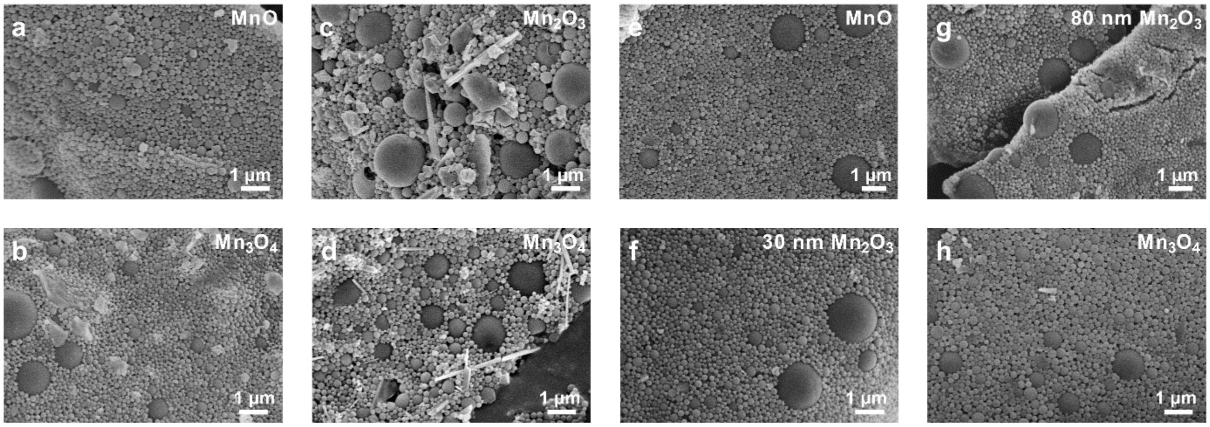

Figure S6. SEM images of PLGA encapsulated In-house (a) MnO and (b) Mn_3O_4 NPs, US Nano's (c) Mn_2O_3 and (d) Mn_3O_4 NPs, and Nanoshel's (e) MnO, (f) Mn_2O_3-30 nm, (g) Mn_2O_3-80 nm, and (h) Mn_3O_4 NPs. Note the long rod-shaped structures present in US Nano's samples along with round-shaped NPs; In-house and Nanoshel's NPs only had spherical shapes.

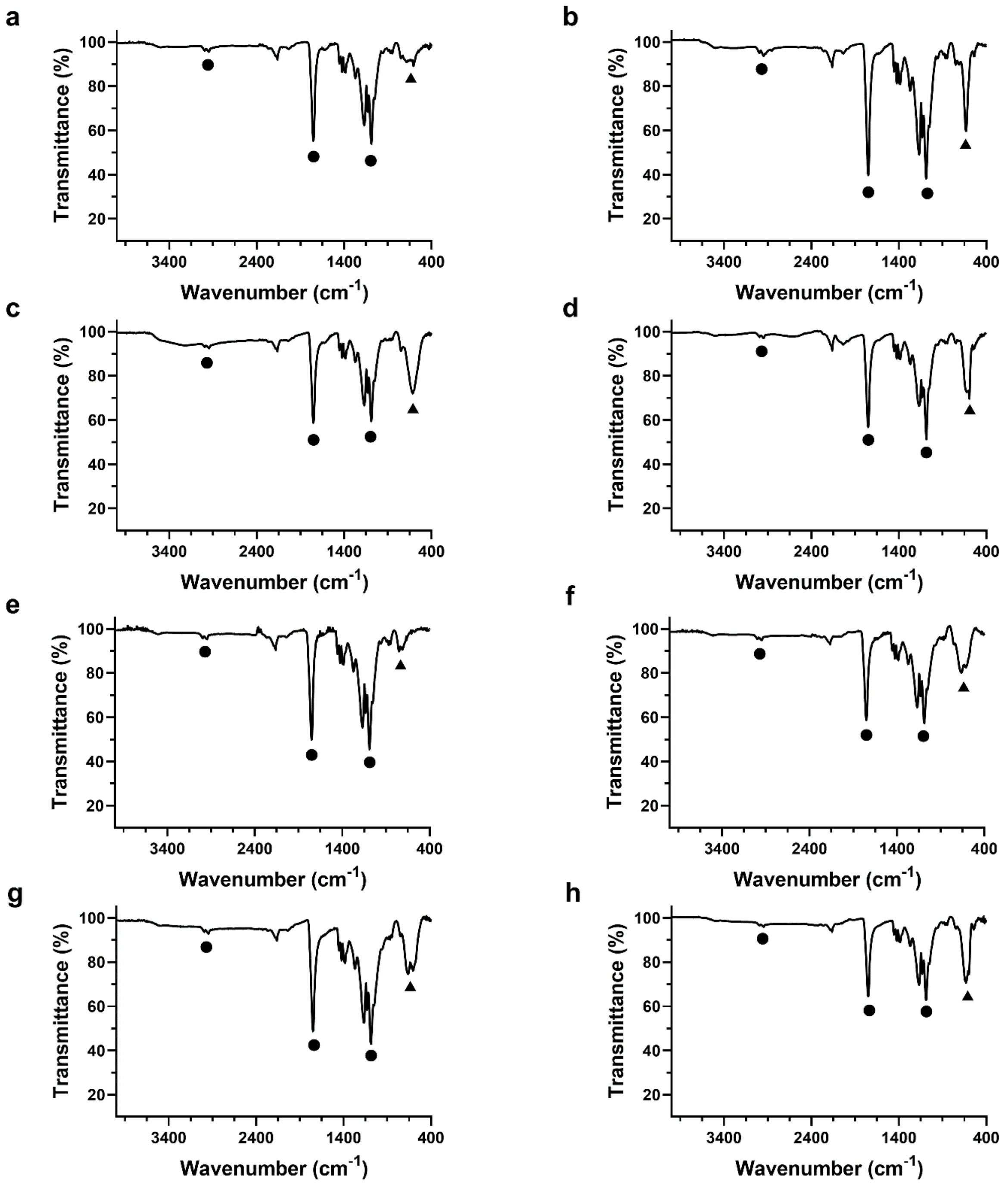

Figure S7. FTIR spectra for Batch #1 of PLGA encapsulated In-house (a) MnO and (b) Mn_3O_4 NPs, US Nano's (c) Mn_2O_3 and (d) Mn_3O_4 NPs, and Nanoshel's (e) MnO, (f) Mn_2O_3-30 nm, (g) Mn_2O_3-80 nm, and (h) Mn_3O_4 NPs. Characteristic peaks for PLGA (●) and manganese oxide (▲) are shown.[4-7] The FTIR spectra was reproducible over the three batches of PLGA encapsulated NPs.

Table S2. Average hydrodynamic size, encapsulation efficiency, and yield for NEMO particles. Values are shown as average +/- St. Dev.

Nanocrystal Type		DLS (nm)	Encapsulation Efficiency (%)	Yield (%)
**In-house**	MnO	183 ± 59	75 ± 21	53 ± 0.1
	Mn_3O_4	267 ± 107	50 ± 6	43 ± 4
**US Nano**	Mn_2O_3	457 ± 223	78 ± 26	53 ± 0.9
	Mn_3O_4	213 ± 71	70 ± 21	55 ± 1
**Nanoshel**	MnO	618 ± 413	74 ± 24	59 ± 3
	Mn_2O_3-30nm	237 ± 34	51 ± 22	59 ± 4
	Mn_2O_3-80nm	491 ± 501	59 ± 7	52 ± 0.1
	Mn_3O_4	164 ± 59	85 ± 27	48 ± 3

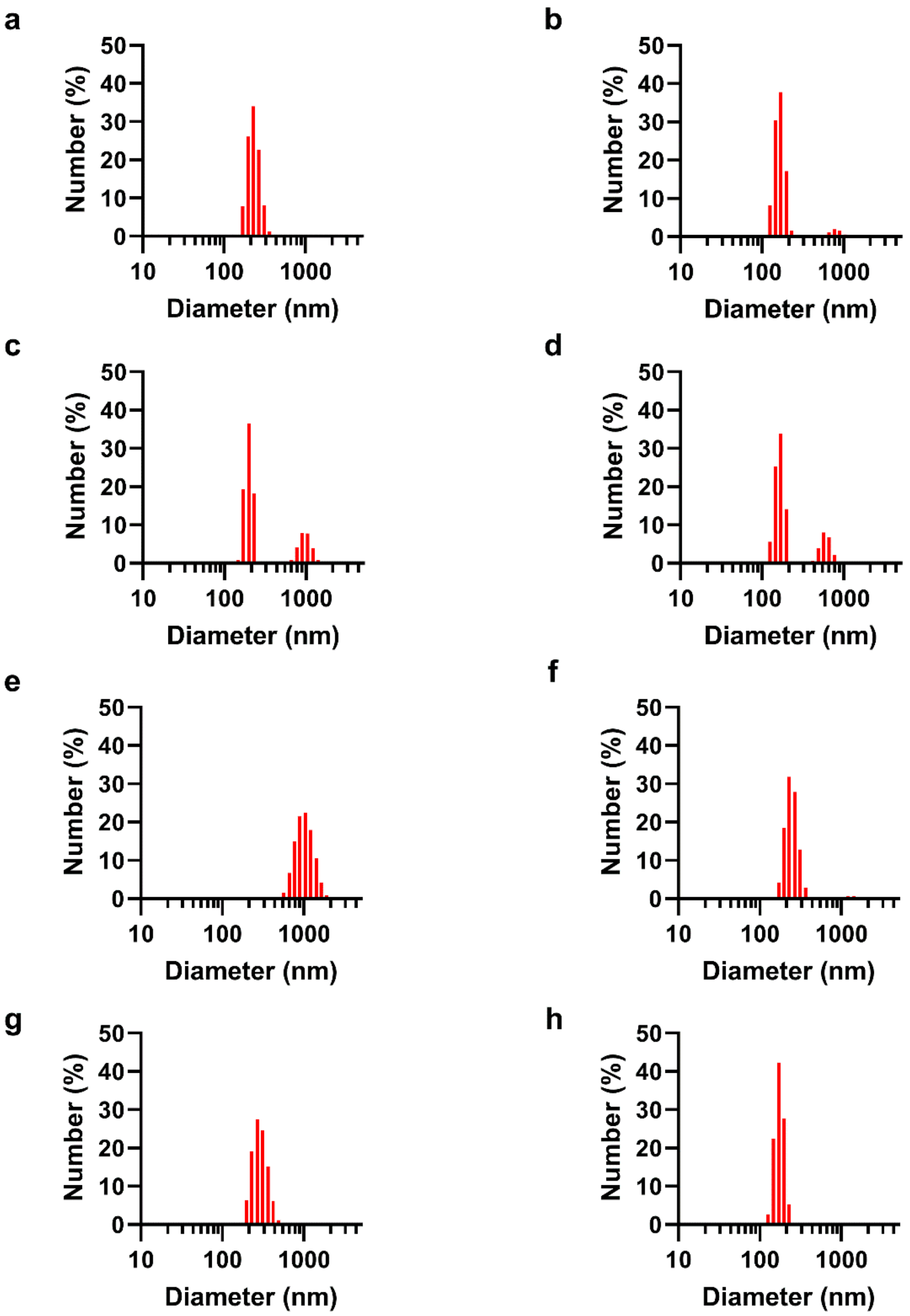

Figure S8. DLS size distribution for Batch #1 of PLGA encapsulated In-house (a) MnO and (b) Mn_3O_4 NPs, US Nano's (c) Mn_2O_3 and (d) Mn_3O_4 NPs, and Nanoshel's (e) MnO, (f) Mn_2O_3-30 nm, (g) Mn_2O_3-80 nm, and (h) Mn_3O_4 NPs.

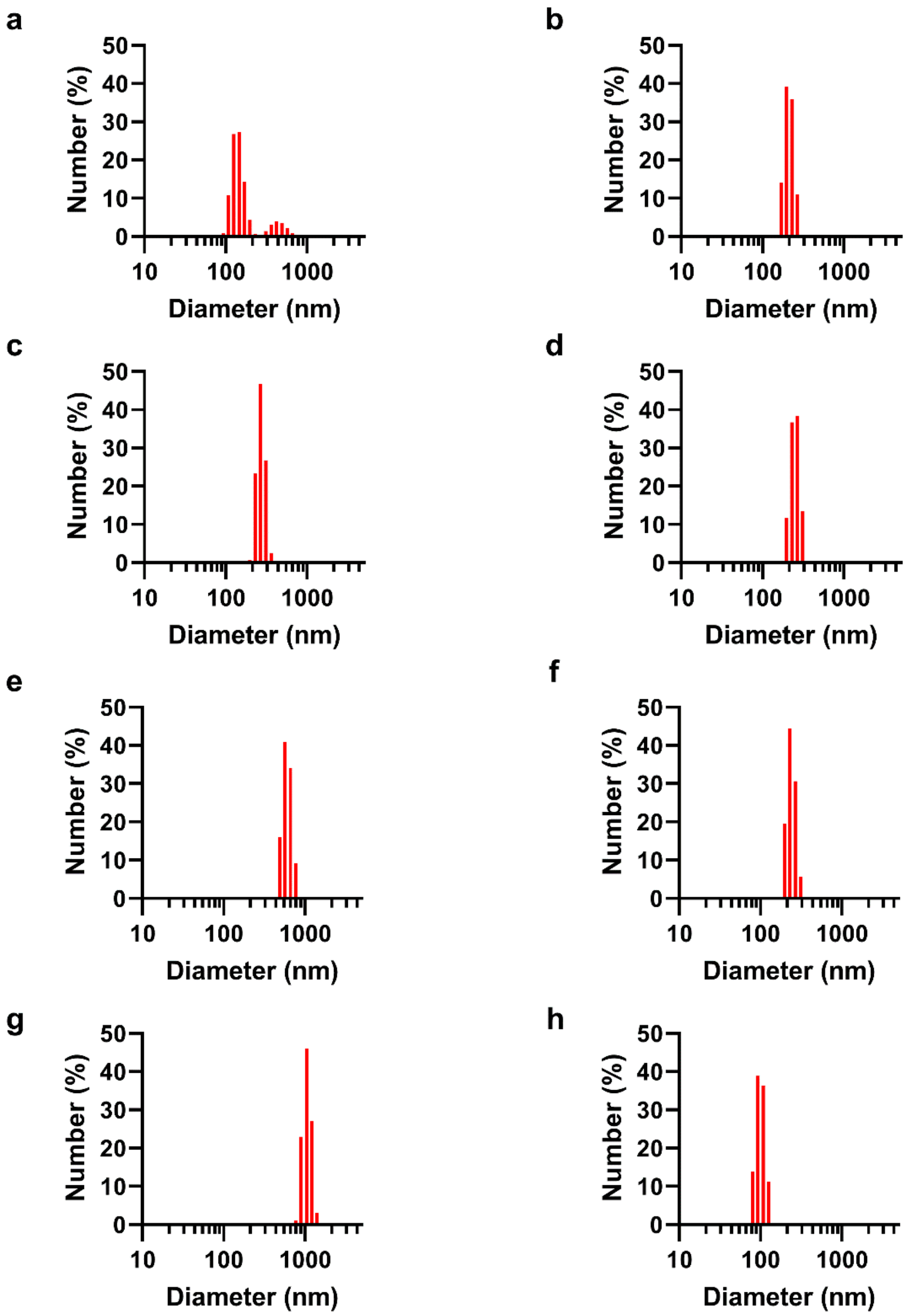

Figure S9. DLS size distribution for Batch #2 of PLGA encapsulated In-house (a) MnO and (b) Mn_3O_4 NPs, US Nano's (c) Mn_2O_3 and (d) Mn_3O_4 NPs, and Nanoshel's (e) MnO, (f) Mn_2O_3-30 nm, (g) Mn_2O_3-80 nm, and (h) Mn_3O_4 NPs.

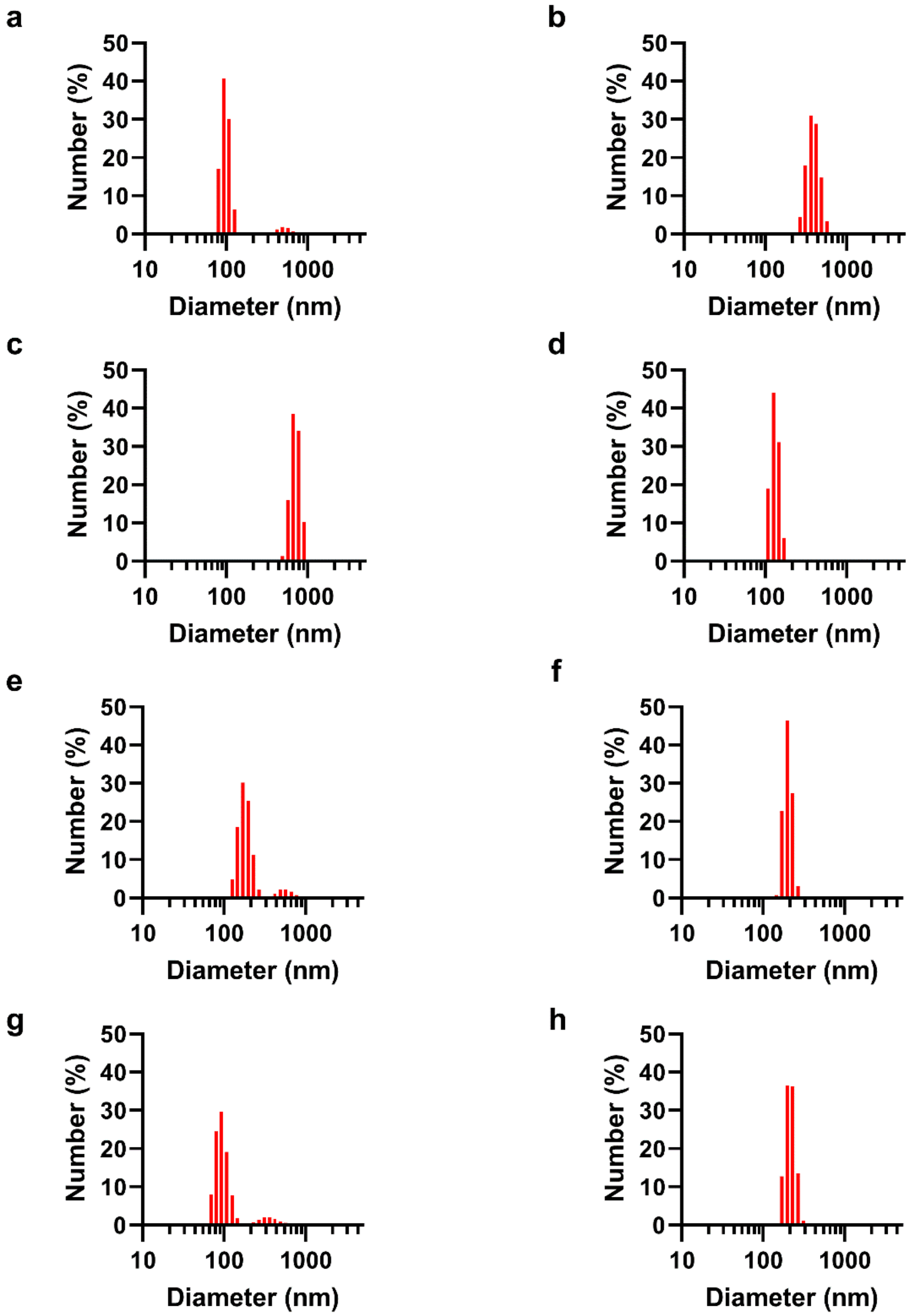

Figure S10. DLS size distribution for Batch #3 of PLGA encapsulated In-house (a) MnO and (b) Mn_3O_4 NPs, US Nano's (c) Mn_2O_3 and (d) Mn_3O_4 NPs, and Nanoshel's (e) MnO, (f) Mn_2O_3-30 nm, (g) Mn_2O_3-80 nm, and (h) Mn_3O_4 NPs.

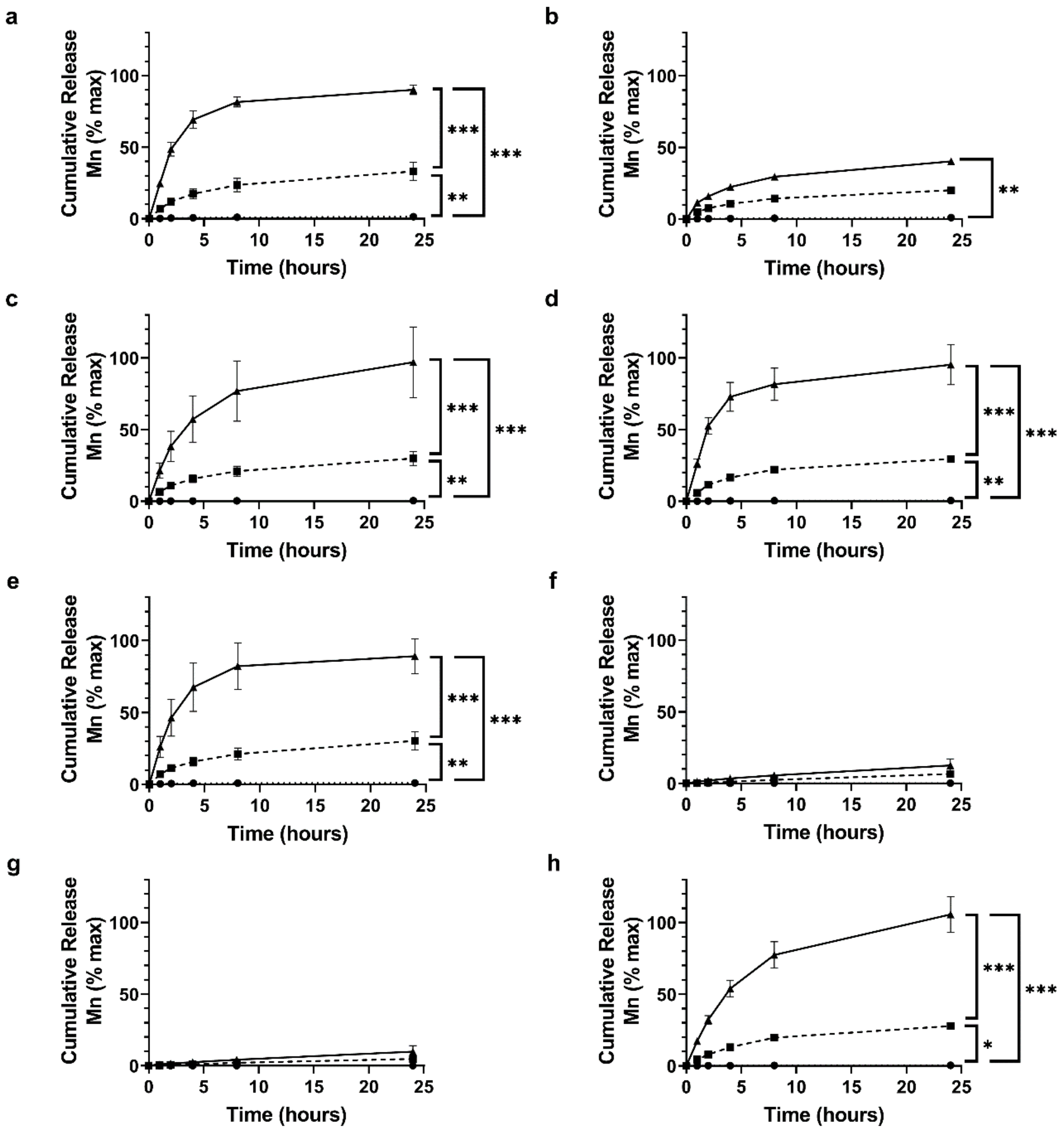

Figure S11. Cumulative release of Mn^{2+} from PLGA encapsulated In-house (a) MnO and (b) Mn_3O_4 NPs, US Nano's (c) Mn_2O_3 and (d) Mn_3O_4 NPs, and Nanoshel's (e) MnO, (f) Mn_2O_3-30 nm, (g) Mn_2O_3-80 nm, and (h) Mn_3O_4 NPs over 24 hr after incubation at pH 7.4 (dotted line), pH 6.5 (dashed line), and pH 5 (solid line). Note the maximal Mn^{2+} release at pH 5 mimicking cell endosomes/lysosomes. Average cumulative release is plotted with standard error of the mean; statistical comparison was made using two-way ANOVA with Holm-Šídák correction. P values are reported as * ≤0.05, ** ≤0.01, *** ≤0.005.

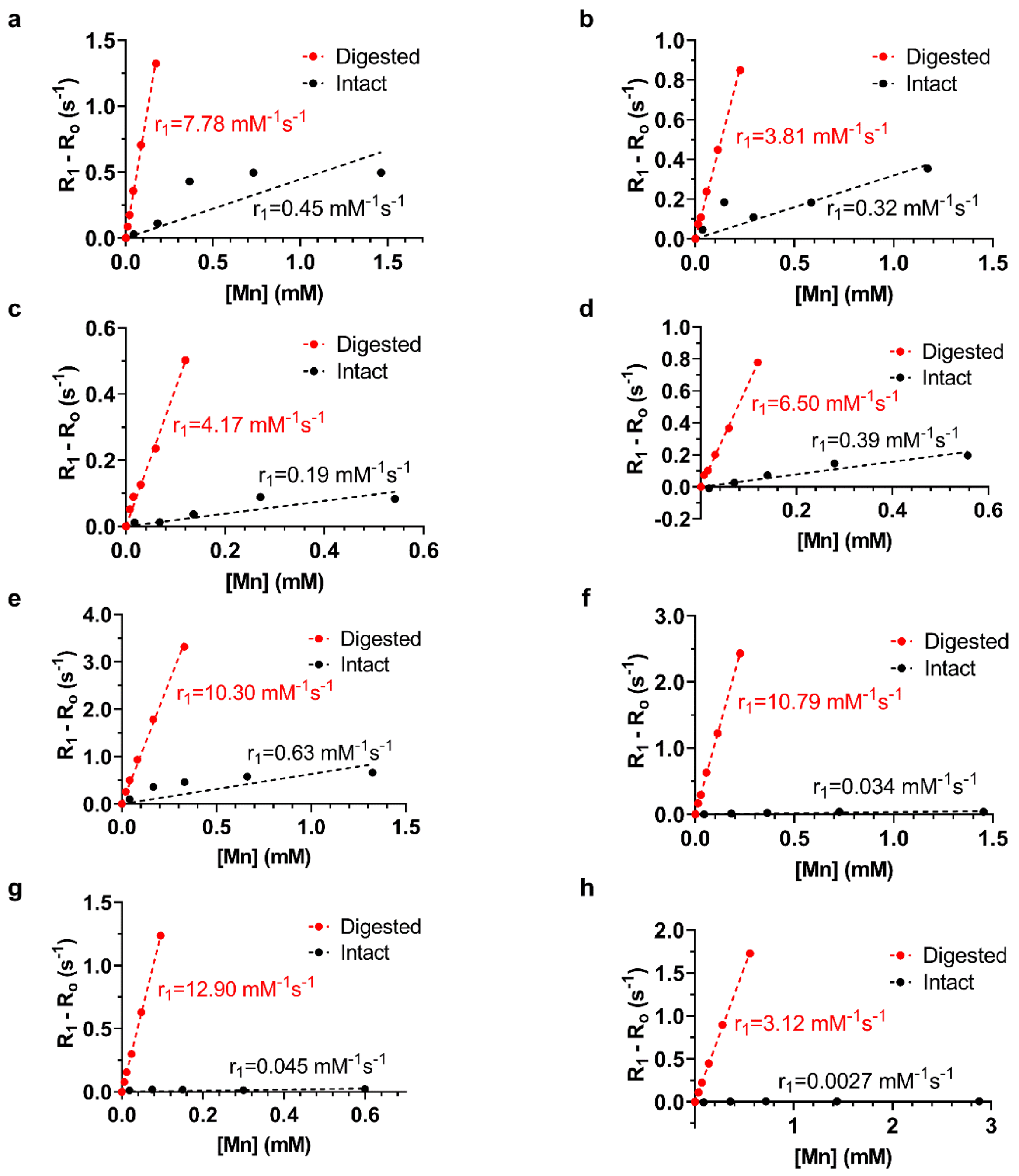

Figure S12. Longitudinal relaxivity r_1 properties of PLGA encapsulated In-house (a) MnO and (b) Mn_3O_4 NPs, US Nano's (c) Mn_2O_3 and (d) Mn_3O_4 NPs, and Nanoshel's (e) MnO, (f) Mn_2O_3-30 nm, (g) Mn_2O_3-80 nm, and (h) Mn_3O_4 NPs**.** Calculated relaxivities (r_1) are shown for NPs either suspended in 0.5% agarose (black) or digested in HCl (red). Digested NPs had much higher r_1 vs. intact NPs.

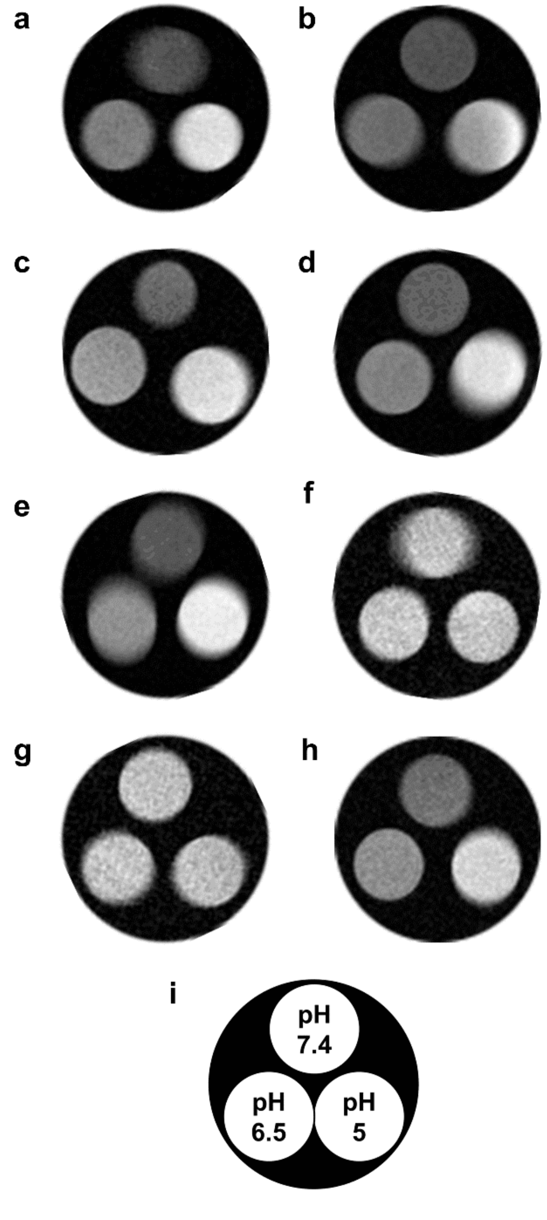

Figure S13. MRI properties of Mn^{2+} supernatants collected from dissolving PLGA encapsulated In-house (a) MnO and (b) Mn_3O_4 NPs, US Nano's (c) Mn_2O_3 and (d) Mn_3O_4 NPs, and Nanoshel's (e) MnO, (f) Mn_2O_3-30 nm, (g) Mn_2O_3-80 nm, and (h) Mn_3O_4 NPs. T_1 MRI images (TR = 400 ms) of supernatants collected from PLGA encapsulated NPs suspended in pH 5, pH 6.5, and pH 7.4 for 1 hr. (i) shows the orientation and placement of tubes in each of the images. Note the brightest signal came from In-house and Nanoshel's MnO NPs incubated at pH 5.

References

1. Martinez de la Torre, C.; Grossman, J. H.; Bobko, A. A.; Bennewitz, M. F., Tuning the size and composition of manganese oxide nanoparticles through varying temperature ramp and aging time. *PloS one* **2020,** *15* (9), e0239034.

2. Mourdikoudis, S.; Liz-Marzán, L. M., Oleylamine in Nanoparticle Synthesis. *Chemistry of Materials* **2013,** *25* (9), 1465-1476.

3. Zheng, M.; Zhang, H.; Gong, X.; Xu, R.; Xiao, Y.; Dong, H.; Liu, X.; Liu, Y., A simple additive-free approach for the synthesis of uniform manganese monoxide nanorods with large specific surface area. *Nanoscale Res Lett* **2013,** *8* (1), 166.

4. Snoderly, H. T.; Freshwater, K. A.; Martinez de la Torre, C.; Panchal, D. M.; Vito, J. N.; Bennewitz, M. F., PEGylation of Metal Oxide Nanoparticles Modulates Neutrophil Extracellular Trap Formation. *Biosensors (Basel)* **2022,** *12* (2).

5. Wang, H.; Zhao, Y.; Wu, Y.; Hu, Y. L.; Nan, K.; Nie, G.; Chen, H., Enhanced anti-tumor efficacy by co-delivery of doxorubicin and paclitaxel with amphiphilic methoxy PEG-PLGA copolymer nanoparticles. *Biomaterials* **2011,** *32* (32), 8281-90.

6. Arasoglu, T.; Derman, S.; Mansuroglu, B., Comparative evaluation of antibacterial activity of caffeic acid phenethyl ester and PLGA nanoparticle formulation by different methods. *Nanotechnology* **2016,** *27* (2), 025103.

7. Jiang, P.; Yu, D.; Zhang, W.; Mao, Z.; Gao, C., Influence of bovine serum albumin coated poly(lactic-co-glycolic acid) particles on differentiation of mesenchymal stem cells. *RSC Advances* **2015,** *5* (51), 40924-40931.
